# Associations between White Meat-Only and Vegetarian Diets with Mortality from All Causes, Heart Diseases, Cancers, and Stroke in the American Cancer Society’s Cancer Prevention Study-II

**DOI:** 10.1016/j.cdnut.2026.109438

**Published:** 2026-07-17

**Authors:** Kelly C Cara, Joan Sabaté, Terryl J Hartman, Alpa V Patel, Caroline Y Um, Marissa M Shams-White

**Affiliations:** 1 Department of Population Science, American Cancer Society, Atlanta, GA, United States; 2 Cancer Prevention and Control Research Program, Winship Cancer Institute, Emory University, Atlanta, GA, United States; 3 Center for Nutrition, Lifestyle, and Disease Prevention, School of Public Health, Loma Linda University, Loma Linda, CA, United States; 4 Department of Epidemiology, Rollins School of Public Health, Emory University, Atlanta, GA, United States

**Keywords:** diet, vegetarian, poultry, red meat, seafood, mortality, heart diseases, neoplasms, stroke, cohort studies

## Abstract

**Background:**

Many dietary guidelines recommend limiting or excluding red and processed meat to promote health and prevent chronic diseases.

**Objectives:**

This study investigated associations between white meat-only and vegetarian diets and mortality from all causes, heart diseases, cancers, and stroke.

**Methods:**

The American Cancer Society’s Cancer Prevention Study-II (CPS-II) cohort participants aged 30 to 95 y at baseline (1982) with mortality data through 2020 were examined (*n =* 998,378; 45% male, *n* = 626,017 deaths). A brief diet questionnaire identified meat intake [red/processed meat intake: high (≥6 d/wk); moderate (4 to <6 d/wk); low (0.5 to <4 d/wk); white meat-only; vegetarian]. Cox proportional hazards models adjusted for demographic, lifestyle, and health factors examined associations between diet categories and mortality risk. Effect modification by sex, smoking, BMI, baseline age group, and attained age was examined.

**Results:**

Most participants consumed red/processed meat (high = 37%, moderate = 31%, low = 31%; white meat-only = 1%; vegetarian = 0.4%). Compared with high red/processed meat intake, low intake was associated with 1% to 5% lower mortality risk from all causes, heart diseases, and cancers across multiple strata, and 4% lower stroke mortality risk overall [hazard ratio (HR): 0.96; 95% confidence interval (CI): 0.94, 0.99]. White meat-only was associated with 5% to 7% lower all-cause mortality risk among males (HR: 0.93; 95% CI: 0.89, 0.98), individuals with healthy weight (HR: 0.95; 95% CI: 0.91, 0.98), and the 50 to 64 y age group (HR: 0.94; 95% CI: 0.90, 0.97), where results were similar with a vegetarian diet (50–64 y; HR: 0.94; 95% CI: 0.88, 1.00). Among participants reaching age ≥80, white meat-only was associated with lower all-cause (HR: 0.94; 95% CI: 0.91, 0.97) and cancer mortality risk (HR: 0.91; 95% CI: 0.83, 0.99). No other associations were statistically significant.

**Conclusions:**

In CPS-II, limiting red/processed meat intake was associated with a small but significantly lower risk of all-cause and cause-specific mortality. Diets excluding red/processed meat may lower risk in certain subgroups, but large studies targeting these diets are needed to elucidate associations with mortality.

## Introduction

Higher dietary intake of red and processed meats has been linked with a greater risk of mortality from all causes, cardiovascular diseases, and related conditions, including stroke, and several cancers [[1], [2], [3], [4], [5]]. Evidence suggests this is due in part to the saturated fat, cholesterol, and heme iron in these meats, along with higher sodium, nitrates, and nitrites in processed meats [2,5]. The International Agency for Research on Cancer has classified processed meats as carcinogenic and red meats as probably carcinogenic to humans [6]. The Dietary Guidelines for Americans (DGA), 2020–2025, cite that dietary patterns relatively lower in red and processed meats are associated with positive health outcomes and recommend replacing processed and high-fat meats with seafood to reduce dietary saturated fat and sodium [7]. Another DGA recommendation is to replace these meats with beans, peas, and lentils to increase dietary fiber. Consumption of red and processed meats and lower dietary fiber intake are independently associated with increased risk of cardiovascular diseases and colorectal cancer [[8], [9], [10]]. In line with this evidence, clinical practice guidelines for cardiovascular diseases and cancers often promote intake of legumes/pulses, lean meat, and fish, while discouraging intake of red and processed meats [11,12].

Several studies have examined associations between consumption of white meat (including poultry and fish) and mortality risk [1,4,13], but the impact on mortality from heart diseases and cancers is still uncertain. A meta-analysis of white meat consumption and mortality in 22 prospective cohorts found a clear inverse association with all-cause mortality, but findings for fatal cardiovascular events were less clear [13]. The World Cancer Research Fund’s 2018 Continuous Update Project reported that evidence for associations between white meat and cancer risk was too limited to draw conclusions [14]. Investigating the impact of excluding all red meat while still consuming white meat could help clarify associations with mortality, but few studies have done this. Additionally, studies examining associations between consuming no meat or fish of any kind (i.e., being vegetarian) and mortality have shown mixed findings across different populations [[15], [16], [17], [18], [19]], so more evidence is still needed. Removing some or all red/processed meats from the diet frees up energy to help meet dietary recommendations for intake of vegetables, fruits, legumes, whole grains, low-fat dairy or nonfat dairy, lean meats and poultry, seafood, nuts, and unsaturated vegetable oils [7]. However, determining associations between white meat-only and vegetarian diets and mortality is challenging because dietary patterns including only white meat or avoiding all meat are rare in many study populations. Furthermore, to study mortality outcomes in populations including generally healthy, non-elderly populations, prospective cohort studies with a lengthy follow-up period are needed [20].

This study aimed to investigate associations between diets completely excluding red and processed meats and risk of mortality from all causes, heart disease, cancers, and stroke in the American Cancer Society’s (ACS) Cancer Prevention Study-II (CPS-II). We hypothesized that, beyond limiting intake of red and processed meats, participants consuming white meat-only or no meat/vegetarian diets would have a lower risk of mortality from all causes and specific causes than those with high intake of red and processed meats.

## Methods

### Study participants

CPS-II is a prospective cohort study including nearly 1.2 million participants (676,306 females and 508,351 males) from all 50 states, the District of Columbia, and Puerto Rico. Details on ACS’s CPS-II cohort study were published previously [21]. Briefly, recruitment began in September 1982, and individuals aged 30 y or older were invited to participate with an emphasis on enrolling older individuals. Participants completed a questionnaire asking about their demographic characteristics; tobacco and alcohol use, diet, and other lifestyle behaviors; personal and family health history; and environmental and occupational exposures. Nearly all questionnaires were returned by November 1982. All aspects of the CPS-II study were approved by the Emory University Institutional Review Board (Atlanta, Georgia).

**FIGURE 1:**
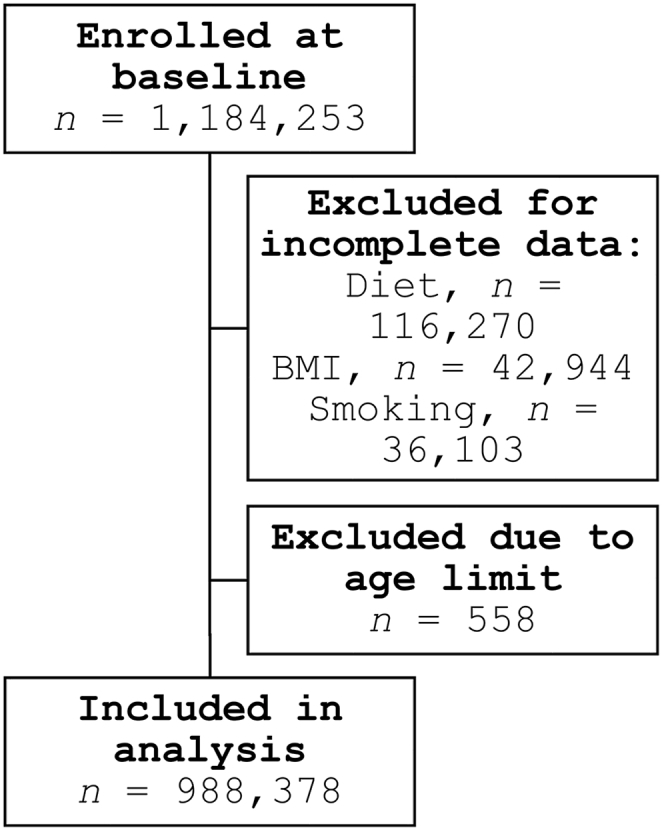
Flow diagram of the analytic cohort selection process.

Participants were excluded from the present study if their baseline data indicated incomplete dietary reporting (*n* = 116,270); missing or implausible weight and height, or a BMI (in kg/m^2^) <18.5 or ≥60 (*n* = 42,944); or incomplete data on smoking status (*n* = 36,103). To allow for sufficient follow-up time, participants aged ≥90 (males) or ≥95 (females) years at baseline were also excluded (*n* = 558). Figure 1 presents the selection process for the final analytic cohort.

### Dietary assessment

At baseline, participants were asked to report the number of days per week, on average, they ate each of 28 different food items, including various meats, fish, dairy foods, eggs, vegetables, fruits, and grain foods. The items were listed in 2 columns on the paper questionnaire. To reduce the likelihood that participants missed a section of the survey, responses were deemed complete only if participants reported 5 or more food items, with ≥1 item reported from each column. The questionnaire did not instruct participants to indicate 0 d/wk for items they did not consume; therefore, if responses were otherwise deemed complete, items left blank were interpreted as “no intake” and re-coded as 0 d/wk of intake. For reporting in descriptive tables, total days per week were summed across items within each food group [range: 0 to (7 × *n* items) d/wk] or reported separately for miscellaneous foods (see Table 1 footnote).

**TABLE 1:** Baseline characteristics^1^ of Cancer Prevention Study-II cohort members included in analysis overall and with comparisons across diet categories^2^

Baseline characteristics	Overall	Diet category				
		No meat/vegetarian	White-meat-only	Red/processed meat—low	Red/processed meat—moderate	Red/processed meat—high
	*n* = 988,378	*n* = 3653	*n* = 8704	*n* = 309,933	*n* = 304,081	*n* = 362,007
Demographics						
Age (y)	56.0 (49.0, 63.0)	60.0 (51.0, 69.0)	60.0 (52.0, 68.0)	58.0 (51.0, 65.0)	56.0 (49.0, 63.0)	54.0 (48.0, 61.0)
Age group						
<50 y	257,856 (26.1)	829 (22.7)	1643 (18.9)	62,904 (20.3)	81,562 (26.8)	110,918 (30.6)
50–64 y	514,981 (52.1)	1503 (41.1)	4125 (47.4)	160,450 (51.8)	160,826 (52.9)	188,077 (52.0)
≥65 y	215,541 (21.8)	1321 (36.2)	2936 (33.7)	86,579 (27.9)	61,693 (20.3)	63,012 (17.4)
Sex, male	442,437 (44.8)	1370 (37.5)	2937 (33.7)	123,352 (39.8)	131,142 (43.1)	183,636 (50.7)
Race/ethnicity						
White or White Hispanic	930,765 (94.2)	3230 (88.4)	7762 (89.2)	288,798 (93.2)	289,539 (95.2)	341,436 (94.3)
Black or Black Hispanic	36,562 (3.7)	269 (7.4)	655 (7.5)	14,281 (4.6)	8759 (2.9)	12,598 (3.5)
Other/missing	21,051 (2.1)	154 (4.2)	287 (3.3)	6854 (2.2)	5783 (1.9)	7973 (2.2)
Education level						
Less than a high school graduate	129,658 (13.1)	677 (18.5)	1575 (18.1)	46,234 (14.9)	33,927 (11.2)	47,245 (13.1)
High school graduate	252,754 (25.6)	638 (17.5)	1799 (20.7)	78,141 (25.2)	77,937 (25.6)	94,239 (26.0)
Vocational/trade school or some college	287,938 (29.1)	1035 (28.3)	2263 (26.0)	87,883 (28.4)	90,405 (29.7)	106,352 (29.4)
College graduate	162,833 (16.5)	587 (16.1)	1283 (14.7)	47,767 (15.4)	52,808 (17.4)	60,388 (16.7)
Graduate school	144,349 (14.6)	609 (16.7)	1539 (17.7)	45,750 (14.8)	46,097 (15.2)	50,354 (13.9)
Missing	10,846 (1.1)	107 (2.9)	245 (2.8)	4158 (1.3)	2907 (1.0)	3429 (0.9)
Marital status						
Married	836,194 (84.6)	2568 (70.3)	5844 (67.1)	243,912 (78.7)	262,276 (86.3)	321,594 (88.8)
Not married	147,314 (14.9)	1038 (28.4)	2755 (31.7)	64,043 (20.7)	40,544 (13.3)	38,934 (10.8)
Missing	4870 (0.5)	47 (1.3)	105 (1.2)	1978 (0.6)	1261 (0.4)	1479 (0.4)
Region of residence						
Midwest	270,122 (27.3)	664 (18.2)	1548 (17.8)	65,104 (21.0)	80,175 (26.4)	122,631 (33.9)
Northeast	201,085 (20.3)	509 (13.9)	2100 (24.1)	74,927 (24.2)	67,572 (22.2)	55,977 (15.5)
South	320,980 (32.5)	1196 (32.7)	2536 (29.1)	101,262 (32.7)	95,399 (31.4)	120,587 (33.3)
West	188,352 (19.1)	1230 (33.7)	2388 (27.4)	65,921 (21.3)	58,732 (19.3)	60,081 (16.6)
Other/missing	7839 (0.8)	54 (1.5)	132 (1.5)	2719 (0.9)	2203 (0.7)	2731 (0.8)
Biometrics and Lifestyle						
BMI (kg/m^2^)	24.8 (22.6, 27.4)	23.9 (21.6, 26.6)	23.8 (21.7, 26.5)	24.5 (22.3, 27.1)	24.7 (22.5, 27.3)	25.1 (22.8, 27.7)
BMI category						
Healthy weight (BMI 18.5 to <25)	509,633 (51.6)	2243 (61.4)	5396 (62.0)	169,122 (54.6)	159,811 (52.6)	173,061 (47.8)
Overweight (BMI 25 to <30)	369,608 (37.4)	1104 (30.2)	2614 (30.0)	110,514 (35.7)	112,365 (37.0)	143,011 (39.5)
Obesity (BMI 30 to <60)	109,137 (11.0)	306 (8.4)	694 (8.0)	30,297 (9.8)	31,905 (10.5)	45,935 (12.7)
Smoking status						
Never smoker	417,041 (42.2)	2297 (62.9)	4186 (48.1)	135,980 (43.9)	128,590 (42.3)	145,988 (40.3)
Former smoker	293,766 (29.7)	777 (21.3)	2751 (31.6)	92,885 (30.0)	90,912 (29.9)	106,441 (29.4)
Ever smoker^3^	59,982 (6.1)	180 (4.9)	539 (6.2)	19,118 (6.2)	17,518 (5.8)	22,627 (6.3)
Current smoker	217,589 (22.0)	399 (10.9)	1228 (14.1)	61,950 (20.0)	67,061 (22.1)	86,951 (24.0)
Alcohol intake (drinks per day)						
Nondrinker	528,257 (53.4)	3072 (84.1)	5867 (67.4)	174,689 (56.4)	156,263 (51.4)	188,366 (52.0)
<1	154,286 (15.6)	174 (4.8)	906 (10.4)	45,078 (14.5)	50,750 (16.7)	57,378 (15.8)
1 to <2	59,078 (6.0)	51 (1.4)	316 (3.6)	16,532 (5.3)	19,930 (6.6)	22,249 (6.1)
2 to <3	42,734 (4.3)	32 (0.9)	160 (1.8)	10,790 (3.5)	14,436 (4.7)	17,316 (4.8)
3 to <4	21,357 (2.2)	18 (0.5)	91 (1.0)	5213 (1.7)	6826 (2.2)	9209 (2.5)
4+	31,320 (3.2)	27 (0.7)	109 (1.3)	7000 (2.3)	9618 (3.2)	14,566 (4.0)
Missing	151,346 (15.3)	279 (7.6)	1255 (14.4)	50,631 (16.3)	46,258 (15.2)	52,923 (14.6)
Amount of physical activity						
None	21,014 (2.1)	95 (2.6)	268 (3.1)	7189 (2.3)	5957 (2.0)	7505 (2.1)
Slight	231,395 (23.4)	682 (18.7)	1572 (18.1)	67,760 (21.9)	73,763 (24.3)	87,618 (24.2)
Moderate	641,639 (64.9)	2336 (63.9)	5643 (64.8)	205,071 (66.2)	198,740 (65.4)	229,849 (63.5)
Heavy	82,344 (8.3)	403 (11.0)	959 (11.0)	25,267 (8.2)	22,285 (7.3)	33,430 (9.2)
Missing	11,986 (1.2)	137 (3.8)	262 (3.0)	4646 (1.5)	3336 (1.1)	3605 (1.0)
Sleep quantity (h/d)						
3–5	37,112 (3.8)	181 (5.0)	489 (5.6)	13,673 (4.4)	10,157 (3.3)	12,612 (3.5)
6	153,992 (15.6)	630 (17.2)	1677 (19.3)	51,048 (16.5)	45,898 (15.1)	54,739 (15.1)
7	332,193 (33.6)	1105 (30.2)	2492 (28.6)	100,871 (32.5)	105,454 (34.7)	122,271 (33.8)
8	380,858 (38.5)	1315 (36.0)	3177 (36.5)	117,457 (37.9)	118,013 (38.8)	140,896 (38.9)
9–14	73,522 (7.4)	281 (7.7)	635 (7.3)	22,549 (7.3)	21,800 (7.2)	28,257 (7.8)
Missing	10,701 (1.1)	141 (3.9)	234 (2.7)	4335 (1.4)	2759 (0.9)	3232 (0.9)
Health history						
Major chronic conditions						
Heart diseases	86,431 (8.7)	347 (9.5)	1169 (13.4)	32,107 (10.4)	25,038 (8.2)	27,770 (7.7)
Cancers (excluding nonmelanoma skin cancers)	66,787 (6.8)	299 (8.2)	752 (8.6)	22,970 (7.4)	20,429 (6.7)	22,337 (6.2)
Stroke	13,611 (1.4)	66 (1.8)	174 (2.0)	4903 (1.6)	3906 (1.3)	4562 (1.3)
Chronic lower respiratory diseases^4^	85,694 (8.7)	299 (8.2)	705 (8.1)	26,009 (8.4)	25,546 (8.4)	33,135 (9.2)
Diabetes	48,653 (4.9)	201 (5.5)	465 (5.3)	15,046 (4.9)	13,552 (4.5)	19,389 (5.4)
Kidney disease	20,265 (2.1)	92 (2.5)	199 (2.3)	6335 (2.0)	5980 (2.0)	7659 (2.1)
Cirrhosis of the liver	2575 (0.3)	13 (0.4)	28 (0.3)	766 (0.2)	690 (0.2)	1078 (0.3)
Total major chronic conditions (out of 7)						
None	724,311 (73.3)	2624 (71.8)	5891 (67.7)	222,175 (71.7)	225,929 (74.3)	267,692 (73.9)
1	214,691 (21.7)	819 (22.4)	2275 (26.1)	70,941 (22.9)	64,028 (21.1)	76,628 (21.2)
2+	49,376 (5.0)	210 (5.7)	538 (6.2)	16,817 (5.4)	14,124 (4.6)	17,687 (4.9)
High blood pressure	280,013 (28.3)	875 (24.0)	2702 (31.0)	94,758 (30.6)	84,303 (27.7)	97,375 (26.9)
Immediate family history of any cancer						
No	509,386 (51.5)	1870 (51.2)	4429 (50.9)	159,098 (51.3)	157,030 (51.6)	186,959 (51.6)
Yes (excluding nonmelanoma skin cancers)	398,408 (40.3)	1386 (37.9)	3348 (38.5)	125,045 (40.3)	123,197 (40.5)	145,432 (40.2)
Yes (site not reported)	50,916 (5.2)	196 (5.4)	445 (5.1)	15,001 (4.8)	15,666 (5.2)	19,608 (5.4)
Missing	29,668 (3.0)	201 (5.5)	482 (5.5)	10,789 (3.5)	8188 (2.7)	10,008 (2.8)
Menopause status (females)						
Premenopausal	104,759 (19.2)	367 (16.1)	812 (14.1)	27,083 (14.5)	35,186 (20.3)	41,311 (23.2)
Perimenopausal	41,379 (7.6)	79 (3.5)	266 (4.6)	11,954 (6.4)	13,661 (7.9)	15,419 (8.6)
Postmenopausal	386,211 (70.7)	1782 (78.1)	4570 (79.2)	143,434 (76.9)	119,713 (69.2)	116,712 (65.4)
Unknown/missing	13,592 (2.5)	55 (2.4)	119 (2.1)	4110 (2.2)	4379 (2.5)	4929 (2.8)
Hormone replacement therapy use (females)						
Never	312,940 (57.3)	1188 (52.0)	2984 (51.7)	101,634 (54.5)	100,620 (58.2)	106,514 (59.7)
Former	101,179 (18.5)	347 (15.2)	989 (17.1)	36,238 (19.4)	32,332 (18.7)	31,273 (17.5)
Current	49,707 (9.1)	144 (6.3)	354 (6.1)	15,377 (8.2)	16,485 (9.5)	17,347 (9.7)
Ever	34,816 (6.4)	180 (7.9)	427 (7.4)	13,057 (7.0)	10,577 (6.1)	10,575 (5.9)
Unknown/missing	47,299 (8.7)	424 (18.6)	1013 (17.6)	20,275 (10.9)	12,925 (7.5)	12,662 (7.1)
Dietary intake^5^						
Total meat/fish	7.5 (6.0, 9.5)	0.0 (0.0, 0.0)	2.5 (1.0, 5.0)	5.0 (3.5, 6.5)	7.0 (6.5, 8.0)	10.0 (8.5, 12.0)
Red/processed meats	5.0 (3.0, 6.5)	0.0 (0.0, 0.0)	0.0 (0.0, 0.0)	2.5 (1.5, 3.0)	5.0 (4.0, 5.0)	7.5 (6.5, 9.0)
White meat	2.0 (1.5, 3.5)	0.0 (0.0, 0.0)	2.5 (1.0, 5.0)	2.0 (1.0, 4.0)	2.5 (1.5, 3.0)	2.0 (1.5, 3.0)
Total dairy foods	6.0 (3.0, 9.0)	2.5 (0.5, 7.0)	2.0 (0.5, 7.0)	4.0 (1.5, 8.0)	6.0 (3.5, 9.0)	7.0 (4.0, 10.5)
Margarine	5.0 (0.5, 7.0)	1.0 (0.0, 7.0)	0.3 (0.0, 5.0)	3.0 (0.3, 7.0)	5.0 (1.0, 7.0)	6.0 (1.0, 7.0)
Eggs	2.0 (1.0, 3.0)	1.0 (0.3, 2.0)	0.5 (0.3, 2.0)	1.0 (0.5, 3.0)	2.0 (1.0, 3.0)	2.0 (1.0, 4.0)
Total vegetables	14.0 (9.5, 18.0)	13.0 (3.0, 20.0)	12.0 (3.0, 19.0)	12.0 (6.5, 17.0)	14.0 (10.0, 18.0)	15.0 (11.0, 19.0)
Non-starchy vegetables	10.0 (6.0, 14.0)	9.0 (1.3, 14.5)	9.5 (2.0, 15.0)	9.0 (4.0, 14.0)	10.0 (7.0, 14.0)	10.0 (7.0, 14.0)
Starchy vegetables	3.5 (2.0, 5.0)	3.0 (0.5, 6.0)	2.0 (0.5, 4.0)	2.5 (1.0, 4.0)	3.5 (2.0, 5.0)	4.5 (3.0, 6.0)
Raw vegetables	2.0 (0.5, 5.0)	2.0 (0.0, 7.0)	2.0 (0.0, 7.0)	2.0 (0.5, 5.0)	2.0 (0.5, 5.0)	2.0 (0.5, 5.0)
Citrus fruits/juices	5.0 (1.0, 7.0)	3.0 (0.3, 7.0)	3.0 (0.3, 7.0)	4.0 (0.5, 7.0)	5.0 (1.0, 7.0)	5.0 (1.0, 7.0)
Total grain foods	9.0 (6.0, 13.0)	8.0 (1.0, 14.0)	7.0 (1.0, 11.5)	8.0 (3.0, 12.0)	9.0 (6.0, 13.0)	10.0 (7.0, 14.0)
Refined grain foods	6.0 (2.5, 9.0)	2.0 (0.5, 6.5)	1.0 (0.3, 6.0)	4.0 (1.5, 7.5)	6.0 (3.0, 9.0)	7.5 (4.0, 9.5)
Whole-grain foods	2.0 (0.5, 6.0)	3.0 (0.3, 8.0)	2.0 (0.0, 7.0)	2.0 (0.5, 6.0)	2.0 (0.5, 6.0)	2.0 (0.5, 6.0)

1 Median (IQR); *n* (%).

2 Comparisons performed with Kruskal–Wallis (continuous characteristics) and Pearson’s *χ*^2^ tests (categorical); *P* < 0.001 for all.

3 Mutually exclusive from all other smokers (i.e., not known if current or former smoker).

4 One condition or more: chronic bronchitis, emphysema, and asthma.

5 Days per week for individual items on the 28-item diet questionnaire (range: 0–7 d/wk) or summed across multiple items [range: 0 to (7 × *n* items) d/wk], as follows. Total meat/fish = red/processed meats (beef + pork + liver + ham + smoked meats + frankfurters/sausage) + white meat (chicken + fish); total dairy foods = butter + cheese + ice cream + chocolate; margarine (individual item); eggs (individual item); total vegetables = non-starchy vegetables (green leafy vegetables + tomatoes + cabbage/broccoli/Brussels sprouts + carrots) + starchy vegetables (squash/corn + potatoes); raw vegetables (individual item); citrus fruits/juices (individual item); total grains = refined grain foods [spaghetti/macaroni/white rice + white bread/rolls/biscuits + cold (dry) cereals] + whole-grain foods (brown rice/whole wheat/barley + bran/corn muffins + oatmeal/shredded wheat/bran cereals).

Among the 28 items listed on the questionnaire were 8 flesh foods, including chicken, fish, and 6 types of red and processed meats (beef, pork, liver, ham, smoked meats, and frankfurters/sausage). Responses to these items were used to group participants into diet categories as follows: “no meat/vegetarian” if they reported consuming flesh foods 0 d/wk or consuming 1 or 2 flesh foods only occasionally (once a month or less); “white-meat-only” if they reported consuming chicken and/or fish but red and processed meats 0 d/wk or consuming 1 or 2 of these meats only occasionally (once a month or less); and red/processed meat consumers. Participants who regularly consumed any red/processed meat (regardless of white meat intake) were categorized into tertiles according to their total frequency of intake across all red and processed meats combined: low (0.5 d/wk to <4 d/wk), moderate (4 d/wk to <6 d/wk), or high (≥6 d/wk).

### Mortality causes

Deaths occurring between the date of enrollment in 1982 and September 1988 were ascertained during biennial personal inquiries by ACS volunteers and verified by death certificates from state health departments. Subsequent deaths through 31 December, 2020 were ascertained through linkage with the National Death Index (NDI) [22]. The present analysis examined mortality from all causes combined and from specific primary causes accounting for ≥200 deaths in each diet category. Major chronic diseases listed among the leading causes of death in the United States in 2023 were considered for analysis [23]. Specific prespecified causes of mortality examined in this study, along with their corresponding International Classification of Diseases (ICD) codes used by the Centers for Disease Control [24], were diseases of the heart (ICD-9 codes: 390-398, 402, 404, and 410-429; ICD-10 codes: I00-I09, I11, I13, and I20-I51), malignant neoplasms/cancers (ICD-9 code: 140-208; ICD-10 code: C00-C97), and cerebrovascular diseases/stroke (ICD-9 code: 430-438; ICD-10 code: I60-I69).

### Covariates

Several potential confounders for the diet and mortality associations examined in this study were identified based on existing literature. These included demographic characteristics, biometrics, lifestyle behaviors, and health history self-reported via baseline surveys. For each measure, participants with missing data were grouped into separate categories to retain the maximum number of participants in analyses. Specific demographic characteristics were 1-y age at interview, sex (male, female), race/ethnicity [White or White Hispanic, Black or Black Hispanic, other/missing (combines “Hispanic,” “Oriental,” and “other”)], education level (less than high school graduate, high school graduate, vocational/trade school or some college, college graduate, graduate school, missing), marital status (married, not married, missing), and region of residence (Midwest, Northeast, South, West, other/missing) as defined by the United States Census Bureau’s Census Regions and Divisions of the United States [25].

Biometrics included self-reported weight and height, which were used to calculate BMI. Participants were categorized by BMI based on NIH and WHO standards [26]. The categories were further divided into low and high levels using thresholds from other CPS-II studies [27,28], as follows: healthy weight-low (18.5 to <22.5), healthy weight-high (22.5 to <25), overweight-low (25 to <27.5), overweight-high (27.5 to <30), obesity-low (30 to <35), obesity-high (35 to <60).

Lifestyle behaviors included 12-category smoking status (never-smokers; former smokers with years since quitting: <10 y, 10 to <20 y, ≥20 y, unknown years since quitting; current smokers with cigarettes per day (CPD) and years of smoking: <20 CPD for <35 y, <20 CPD for ≥35 y, ≥20 CPD for <35 y, ≥20 CPD for ≥35 y, unknown CPD or duration; ever cigarette smokers, status unknown; ever pipe/cigar smokers without cigarettes), where pipe/cigar status was only captured in males, and “ever-smokers” were mutually exclusive from all other smokers. Other lifestyle behaviors were alcohol intake [drinks per day: nondrinkers (combines “0 drinks per day” and blank responses), <1, 1 to <2, 2 to <3, 3 to <4, ≥4, missing], amount of physical activity (none, slight, moderate, heavy, missing), and sleep quantity (hours per day: 3–5, 6, 7, 8, 9–14, missing). Reports of average sleep of <3 h or >14 h were deemed unlikely and re-coded as missing.

Baseline health history was used to create binary variables (yes, no) to identify participants with the following major chronic conditions: heart disease, cancers (excluding nonmelanoma skin cancers), stroke, one or more chronic lower respiratory diseases (chronic bronchitis, emphysema, and asthma), diabetes, kidney disease, and cirrhosis of the liver. Participants were then categorized based on their total number of major conditions (none, 1, 2+). Participants also reported whether they had ever been diagnosed with high blood pressure (yes, no). Family history of cancer was reported for immediate family members (i.e., either parent and any sibling) and was categorized as follows: no, yes (excluding nonmelanoma skin cancers), yes (site not reported), or missing. For females, health history also included menopause status (premenopausal, perimenopausal, postmenopausal, unknown/missing) and hormone replacement therapy use (never, former, current, ever, unknown/missing), where males were grouped with premenopausal females and never-users of hormone replacement therapy in analyses not stratified by sex.

### Statistical analyses

Follow-up time began on the date participants returned the baseline questionnaire in 1982 and ended on the first occurring date from: the date they died, the study end date of 31 December, 2020, or 1 September, 1988, if their identifying data were insufficient for NDI linkage (0.2% of the analytic cohort). To address potential ghost-time bias [29], cohort members without a recorded death date prior to the end of the study were censored on the date they turned age 90 (males; 8.6% of the analytic cohort) or 95 (females; 6.1% of the analytic cohort). Cox proportional hazards models were performed to determine hazard ratios (HR) and 95% confidence intervals (CI) for mortality across follow-up time in continuous years using the "survival" package in R [30]. For all models, the primary exposure was diet category, where participants with high intake of red/processed meat (≥6 d/wk) served as the reference group. In unadjusted models for diet category with each mortality outcome, proportional hazards were checked statistically and visually with Schoenfeld residuals, log–log plots, and Kaplan–Meier curves. For some outcomes, proportional hazards were violated statistically, but visual inspection indicated hazards were qualitatively similar over time. Therefore, the results presented below include the full follow-up period.

To account for natural variations in mortality risk by age, all adjusted models were stratified by 1-y age at baseline (model 1: age-adjusted). Subsequent models serially added covariate groups, as follows. Model 2 added demographic characteristics (sex, race/ethnicity, education level, marital status, and region of living). Model 3 added lifestyle behaviors (smoking status, alcohol intake, physical activity level, and sleep quantity). Model 4 added health history variables tailored for each mortality outcome (e.g., for all-cause mortality, models adjusted for number of comorbidities and high blood pressure; see Supplemental Table 1-9 footnotes for specific health history adjustments). Model 5 added the BMI category (fully adjusted).

We tested for effect modification by checking interactions between diet and sex (male, female), BMI category (healthy weight, overweight, obesity), smoking status (never, former, ever, current), education level (college or higher: yes/no), baseline health status (healthy: yes/no), baseline age group [<50 y (born in 1932–1952); 50–64 y (born in 1918–1932); ≥65 y (born in 1917 or earlier)], and age attained by the end of follow-up (<80 y, ≥80 y). The age group analysis examined birth cohort effects, whereas the attained age analysis examined life expectancy below or above the analytic cohort’s median age of 80 y at the time of mortality. Where significant interactions were identified, we stratified analyses by subgroups if case counts were sufficient. In BMI-stratified analyses, continuous BMI was added to the fully adjusted models in place of the BMI category. For smoking-stratified analyses, the lifestyle-adjusted models replaced smoking status with years since quitting (former smoker strata) and CPD plus duration of smoking (current smoker strata).

To reduce the potential for reverse causality, we conducted sensitivity analyses excluding those with a history of any major comorbidities listed above (i.e., healthy cohort analysis), and we excluded participants with <2 y of follow-up (e.g., deaths occurring in the first 2 y). To further investigate the all-cause mortality findings and address potential residual confounding due to smoking, an additional analysis was performed among never-smokers and former smokers with 20 or more years since quitting (i.e., long-term nonsmokers). Due to limitations of the baseline diet survey, other important dietary factors [i.e., intake (days per week)] of total vegetables, whole grains, and citrus fruit/juices) were not adequately captured so were adjusted only in a sensitivity model. Finally, to explore whether white meat may mitigate the deleterious effects of red meat in a mixed diet, we conducted a post hoc exploratory analysis comparing participants reporting red meat-only, white meat-only, and no meat/vegetarian diets to those reporting mixed diets with red and white meat intake (any days per week). All analyses were performed using R Statistical Software [31], and statistical significance was determined by CI that did not include 1.00 or *P* < 0.05.

## Results

### Participant characteristics

Of the nearly 1.2 million original CPS-II baseline cohort members, 84% were included in this study (*n* = 988,378; 45% male, 94% White or White Hispanic). Sociodemographic, lifestyle, and health characteristics of the analytical sample closely resembled those of the full cohort (data not shown). The median (IQR) age at baseline was 56 (49, 63) years, and the average follow-up time was 27.5 (17.0, 37.1) years with a maximum of 38.5 y. Table 1 presents baseline characteristics for the study participants overall and by diet category. The majority of study participants reported regular intake of red and processed meats, with 37% reporting high intake, 31% reporting moderate intake, and 31% reporting low intake. Of these, 1.3% reported consuming only red/processed meats with no white meat intake. Just 0.9% of participants reported consuming white meat-only, and 0.4% reported no regular meat intake of any kind (i.e., no meat/vegetarian). Compared to participants in the red/processed meat groups, those in the white meat-only and vegetarian groups were slightly older at baseline, more likely to be female, less likely to be White or White Hispanic, and more likely to have less than a high school diploma or to have attended graduate school. They were also less likely to be married and more likely to be from the Western United States region.

Median BMI was similar across all diet categories. Nearly two-thirds of the white meat-only and vegetarian participants had a healthy weight (62% and 61%, respectively), and obesity rates were lowest in these groups (8% for both). Among those consuming red/processed meat, those with low intake were most likely to have a healthy weight (low: 55%; moderate: 53%; high: 48%), whereas those with high intake were most likely to have obesity (low: 10%; moderate: 10%; high: 13%). Across all diet categories, the largest proportion of participants were never-smokers and nondrinkers, but more vegetarians had never smoked (63%) and were nondrinkers (84%) compared to participants from any other group. Amount of physical activity was similar across diet categories, with slightly more participants in the white meat-only and vegetarian groups reporting “heavy” physical activity and more participants from the red/processed meat groups reporting “slight” physical activity. Those in the white meat-only and vegetarian groups were more likely to report sleeping an average of ≤6 h/d. Most participants reported no major chronic conditions at baseline. Compared to all other groups, a greater proportion of the white meat-only group had one or more major conditions (32%).

Dietary intake varied across the diet categories. Participants in the high red/processed meat group reported the highest average intake for total meat/fish, total dairy foods, margarine, total and starchy vegetables, and total and refined grain foods. The high and moderate red/processed meat groups had greater intakes of eggs, non-starchy vegetables, and citrus fruits/juices, whereas the vegetarian group reported a higher average intake of whole-grain foods. Those in the white meat-only group reported the lowest average intake for total dairy foods, margarine, eggs, starchy vegetables, total grain foods, and refined grain foods. The white meat-only and vegetarian groups had the lowest intakes of citrus fruits/juices.

In this study, 626,017 deaths were observed by the end of follow-up, accounting for 63% of study participants over 25,351,291 total person-years. Table 2 presents frequencies for deaths from all causes and specific causes in the full analytic cohort and by diet category. Major chronic conditions accounted for 74% of deaths in the analytic cohort, and patterns across conditions resembled those of the United States population [23]. Heart diseases and cancers were the leading causes of mortality, where trachea, bronchus, and lung cancers accounted for 24% of all cancer deaths, followed by colon (9%), breast (8%), prostate (7%), and pancreatic cancers (7%).

**TABLE 2:** Mortality from all causes and specific causes^1^ for Cancer Prevention Study-II cohort members included in analysis overall and with comparisons across diet categories^2^

Cause of mortality	Overall	Diet category				
		No meat/vegetarian	White meat-only	Red/processed meat—low	Red/processed meat—moderate	Red/processed meat—high
	*n* = 988,378	*n* = 3653	*n* = 8704	*n* = 309,933	*n* = 304,081	*n* = 362,007
All causes	626,017 (63.3)	2197 (60.1)	5495 (63.1)	201,402 (65.0)	190,140 (62.5)	226,783 (62.6)
Specific causes						
Major chronic conditions						
Heart diseases	178,469 (18.1)	678 (18.6)	1754 (20.2)	60,033 (19.4)	53,136 (17.5)	62,868 (17.4)
Cancers	161,992 (16.4)	512 (14.0)	1280 (14.7)	49,590 (16.0)	49,811 (16.4)	60,799 (16.8)
Stroke	43,750 (4.4)	200 (5.5)	420 (4.8)	14,697 (4.7)	13,421 (4.4)	15,012 (4.1)
Chronic lower respiratory diseases	29,806 (3.0)	66 (1.8)	197 (2.3)	8923 (2.9)	9019 (3.0)	11,601 (3.2)
Alzheimer’s disease	23,088 (2.3)	80 (2.2)	194 (2.2)	7462 (2.4)	7279 (2.4)	8073 (2.2)
Diabetes	13,512 (1.4)	34 (0.9)	95 (1.1)	4066 (1.3)	3820 (1.3)	5497 (1.5)
Kidney diseases	8971 (0.9)	29 (0.8)	75 (0.9)	2898 (0.9)	2648 (0.9)	3321 (0.9)
Liver diseases	3572 (0.4)	12 (0.3)	22 (0.3)	1099 (0.4)	1073 (0.4)	1366 (0.4)
All other causes	162,857 (16.5)	586 (16.0)	1458 (16.8)	52,634 (17.0)	49,933 (16.4)	58,246 (16.1)
Not dead	362,361 (36.7)	1456 (39.9)	3209 (36.9)	108,531 (35.0)	113,941 (37.5)	135,224 (37.4)

1 *n* (%).

2 Comparisons were performed with Pearson’s *χ*^2^ tests; *P* < 0.001 for all causes and specific causes.

### Associations between diet categories and mortality from all causes and specific causes

**FIGURE 2:**
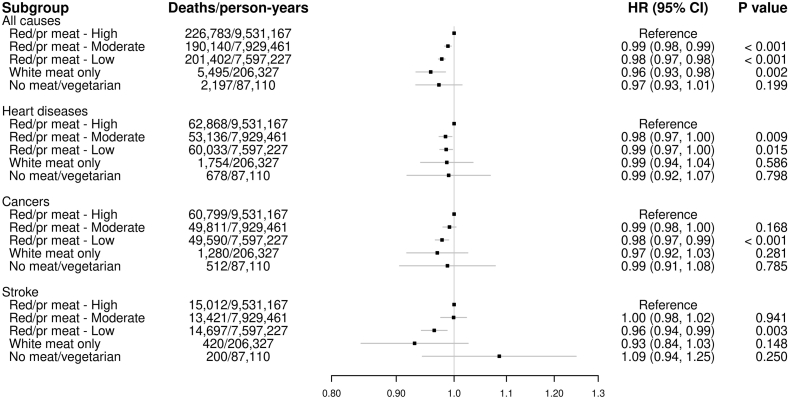
Risk of all-cause and cause-specific mortality across diet categories based on fully adjusted Cox proportional hazards models. Model adjustments included age, demographic factors (sex, race, education level, marital status, United States Census Bureau region), lifestyle factors (12-level smoking status, alcohol intake, physical activity, sleep), and health history (unique covariates for each mortality outcome were as follows. *All causes*: number of comorbidities, high blood pressure; *heart diseases:* history of heart disease, high blood pressure, menopause status, hormone replacement therapy; *cancers*: history of cancer, family history of cancer, history of diabetes, menopause status, hormone replacement therapy; *stroke*: history of stroke, high blood pressure, history of diabetes, menopause status, hormone replacement therapy, and 6-level BMI. CI, confidence interval; HR, hazard ratio; pr, processed.

Age-adjusted Cox models showed a dose–response relationship between diet and mortality from all causes, heart diseases, cancers, and stroke, but most associations were attenuated in fully adjusted models (Supplemental Table 1). Significant associations with all mortality outcomes persisted only for the low red/processed meat group. Figure 2 presents results from fully adjusted models for all-cause and cause-specific mortality. The effect of diet on all-cause, heart disease, and cancer mortality showed significant modification by multiple factors, and stratified results from fully adjusted models for these outcomes are described below. The effect of diet on stroke mortality was not modified by any factor tested. In the fully adjusted model, only low red/processed meat intake was associated with significantly lower risk of stroke mortality compared to high intake (HR: 0.96; 95% CI: 0.94, 0.99).

#### All-cause mortality

**FIGURE 3:**
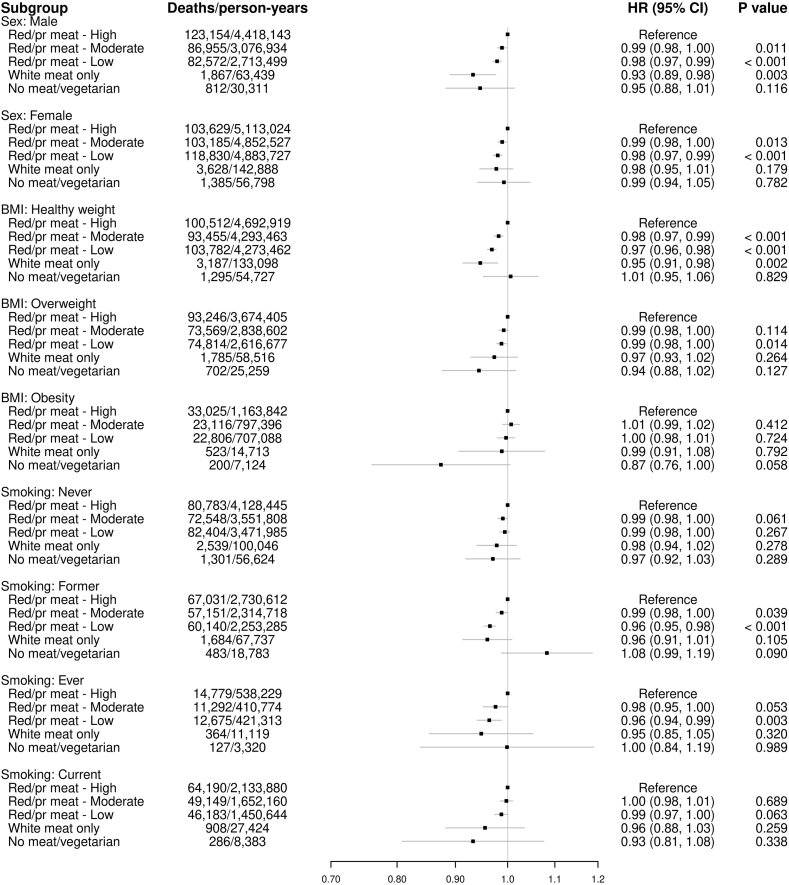
Risk of all-cause mortality across diet categories based on fully adjusted Cox proportional hazards models stratified by sex, BMI, and smoking status. Model adjustments included age, demographic factors [sex (not adjusted in sex strata), race, education level, marital status, United States Census Bureau region], lifestyle factors [12-level smoking status (not adjusted in smoking strata; replaced with years since quit (*former smokers*) or cigarettes per day with years (*current smokers*), alcohol intake, physical activity, sleep], health history (number of comorbidities, high blood pressure), and 6-level BMI (continuous BMI for BMI strata). CI, confidence interval; HR, hazard ratio; pr, processed.

**FIGURE 4:**
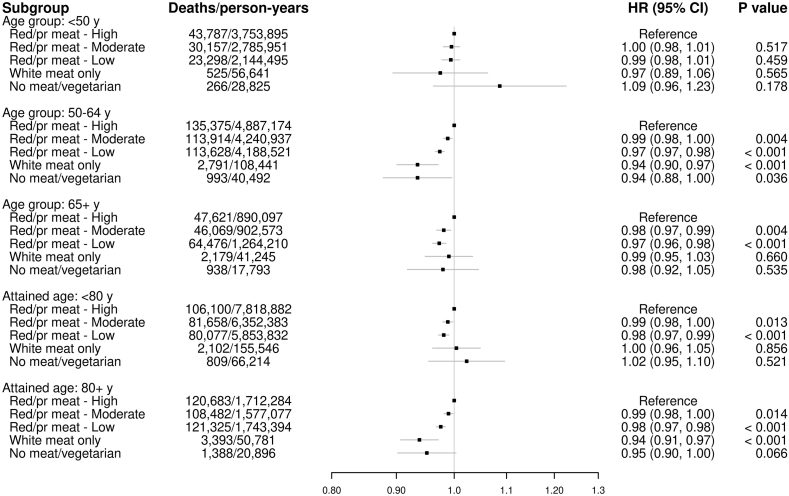
Risk of all-cause mortality across diet categories based on fully adjusted Cox proportional hazards models stratified by baseline age group and attained age. Model adjustments included age, demographic factors (sex, race, education level, marital status, United States Census Bureau region), lifestyle factors (12-level smoking status, alcohol intake, physical activity, sleep), health history (number of comorbidities, high blood pressure), and 6-level BMI. CI, confidence interval; HR, hazard ratio; pr, processed.

The effect of diet on all-cause mortality showed significant interactions with sex, BMI category, smoking status, age group, and attained age (Supplemental Table 2). Figure 3 depicts results stratified by sex, BMI, and smoking, and Figure 4 depicts the age group and attained age results. Compared with high red/processed meat intake, moderate and low intakes were associated with significantly lower risk of all-cause mortality among both males and females, participants with a healthy weight, former smokers, those in the 50 to 64 y and ≥65 y age groups, and those who attained an age of <80 y or ≥80 y during follow-up. White meat-only intake was associated with a further decrease in all-cause mortality risk among males, participants with a healthy weight, the 50 to 64 y age group, and those who attained an age of ≥80 y. Low red/processed meat intake was additionally associated with significantly lower all-cause mortality risk among participants with an overweight BMI and among ever-smokers. Similar to findings for white meat-only, a vegetarian diet was associated with 6% lower risk of all-cause mortality in the 50 to 64 y age group (HR: 0.94; 95% CI: 0.88, 1.00). All other associations among vegetarians were nonsignificant.

#### CVD mortality

**FIGURE 5:**
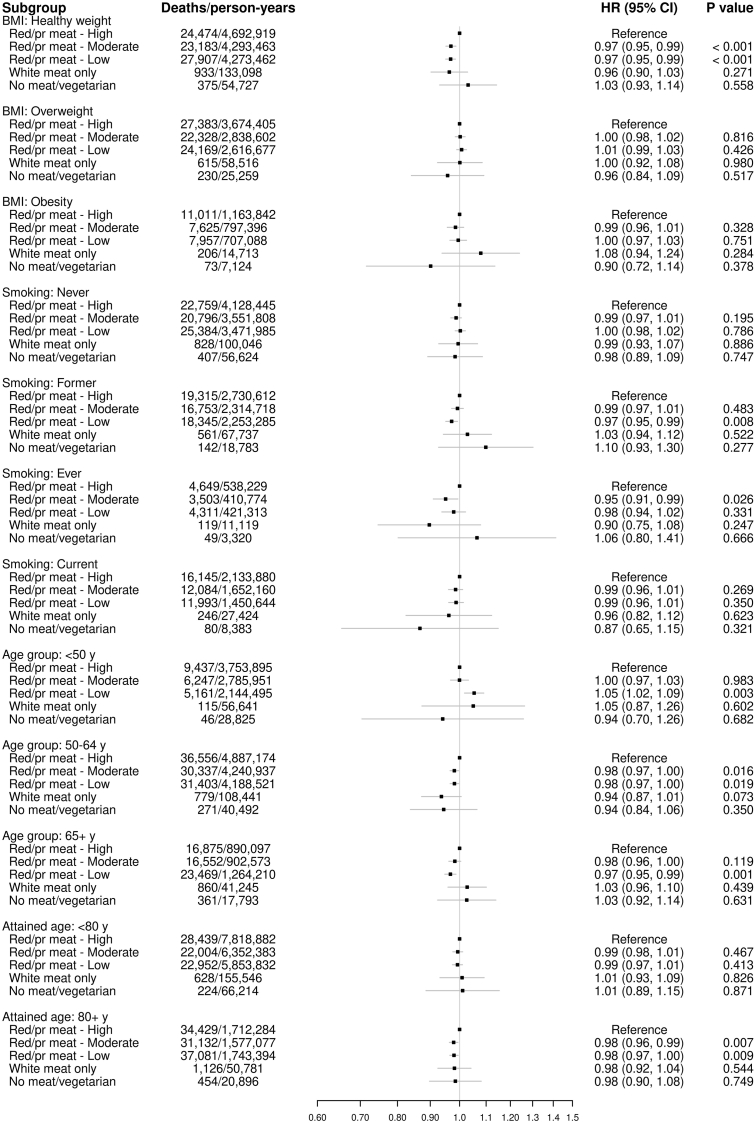
Risk of heart disease mortality across diet categories based on fully adjusted Cox proportional hazards models stratified by BMI, smoking status, baseline age group, and attained age. Model adjustments included age, demographic factors (sex, race, education level, marital status, United States Census Bureau region), lifestyle factors [12-level smoking status (not adjusted in smoking strata; replaced with years since quit (*Former smokers*) or cigarettes per day with years (*Current smokers*), alcohol intake, physical activity, sleep], health history (history of heart disease, high blood pressure, menopause status, hormone replacement therapy), and 6-level BMI (continuous BMI for BMI strata). CI, confidence interval; HR, hazard ratio; pr, processed.

The effect of diet on heart disease mortality showed significant interactions with BMI category, smoking status, age group, and attained age. Results from fully adjusted Cox models stratified by these factors are presented in Supplemental Table 2 and Figure 5. Compared with high intake of red/processed meat, moderate and low intakes were significantly associated with lower heart disease mortality risk among participants with healthy weight, former smokers (low intake only), ever-smokers (moderate intake only), the 50 to 64 y age group, the ≥65 y age group (low intake only), and those reaching ≥80 y by the end of follow-up. Low red/processed meat intake was associated with significantly higher risk of heart disease mortality in the <50 y age group. Intake of white meat-only and vegetarian diets showed no significant associations with heart disease mortality.

#### Cancer mortality

**FIGURE 6:**
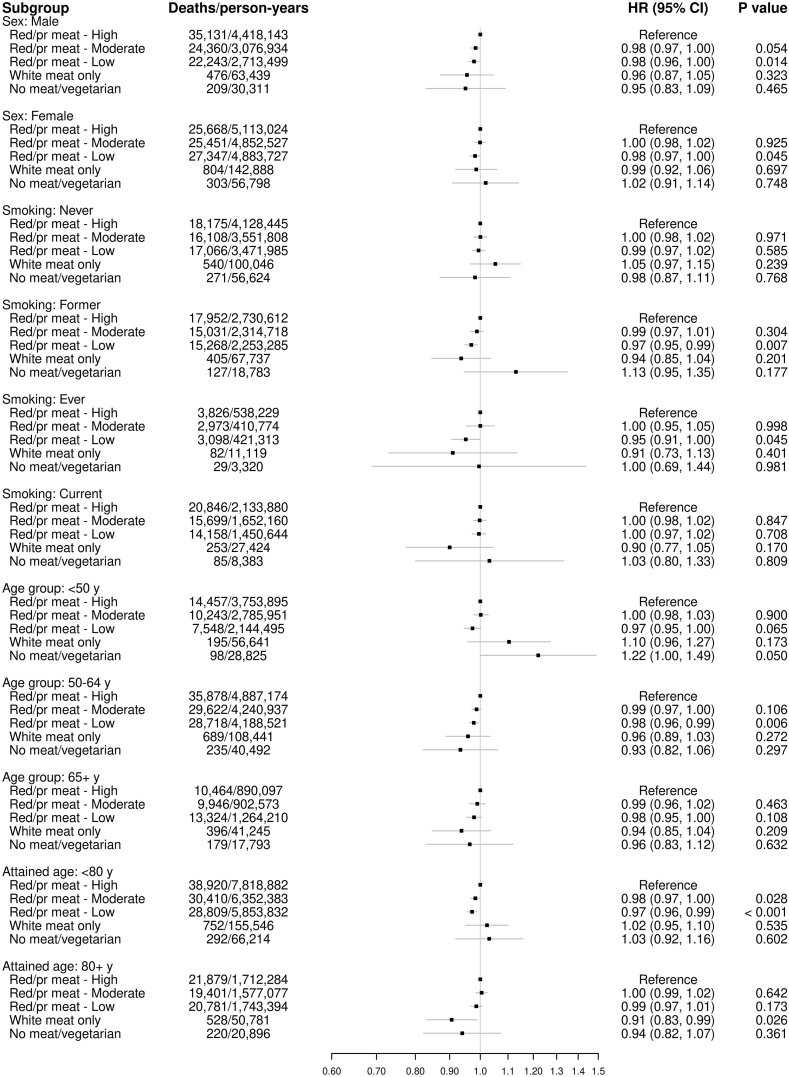
Risk of cancer mortality based on fully adjusted Cox proportional hazards models stratified by sex, smoking status, baseline age group, and attained age. Model adjustments included age, demographic factors [sex (not adjusted in sex strata), race, education level, marital status, United States Census Bureau region], lifestyle factors [12-level smoking status (not adjusted in smoking strata; replaced with years since quit (*Former smokers*) or cigarettes per day with years (*Current smokers*), alcohol intake, physical activity, sleep], health history (history of cancer, family history of cancer, history of diabetes, menopause status, hormone replacement therapy), and 6-level BMI. CI, confidence interval; HR, hazard ratio; pr, processed.

The effect of diet on cancer mortality showed significant interactions with sex, smoking status, age group, and attained age. Results from fully adjusted Cox models stratified by these factors are presented in Figure 6 and Supplemental Table 2. Among most strata, only low intake of red/processed meat showed significant associations with cancer mortality risk. Compared to high red/processed meat intake, low intake was associated with 5% lower cancer mortality risk among ever-smokers (HR: 0.95; 95% CI: 0.91, 1.00), whereas risk was 2% to 3% lower among males and females, former smokers, the 50 to 64 y age group, and those with an attained age <80 y. Moderate red/processed meat intake was also associated with lower cancer mortality risk in those with an attained age <80 y, and those reaching ≥80 y showed significantly lower risk if consuming white meat-only. A vegetarian diet was not associated with risk of cancer mortality.

### Sensitivity analyses

When dietary components were added to the fully adjusted model, effects did not change appreciably from those of the main analysis (Supplemental Table 3). In the healthy cohort sensitivity analysis, results from fully adjusted Cox models largely resembled those of the main analyses for overall effects (Supplemental Table 3) and stratified effects (Supplemental Tables 4–7) for all-cause and cause-specific mortality. However, there were notable changes in the magnitude and significance of some associations and interactions. Compared to high red/processed meat intake, the association between consuming white meat-only and all-cause mortality became nonsignificant in males (HR: 0.96; 95% CI: 0.90, 1.02) and significant in females (HR: 0.96; 95% CI: 0.92, 1.00). A vegetarian diet became significantly associated with a 20% lower risk of all-cause mortality among participants with obesity (HR: 0.80; 95% CI: 0.66, 0.96) and a 19% higher risk among former smokers (HR: 1.19; 95% CI: 1.06, 1.34). White meat-only intake became associated with 11% lower risk of all-cause mortality in those <50 y (HR: 0.89; 95% CI: 0.80, 0.99), whereas risk associated with a vegetarian diet was attenuated in the 50 to 64 y age group (HR: 0.98; 95% CI: 0.91, 1.05). The effect of diet on stroke mortality risk showed a significant interaction by sex. Among males, a vegetarian diet was associated with a 50% higher risk of stroke mortality compared to high red/processed meat intake (HR: 1.50; 95% CI: 1.14, 1.97), though there were few cases in this group.

In the sensitivity analysis excluding the first 2 y of follow-up, overall results across mortality outcomes were nearly identical to the main analyses, with some shifts in statistical significance (Supplemental Tables 3–7). Notably, a vegetarian diet became associated with significantly higher all-cause mortality risk among former smokers and higher cancer mortality risk in the <50 y age group than high red/processed meat intake. Moderate and low red/processed meat intake became significantly associated with lower all-cause mortality risk among ever-smokers and current smokers, respectively. The effect of diet on stroke mortality showed a significant interaction with smoking status; however, due to low case numbers among ever-smokers and current smokers, analyses focused on never-smokers and former smokers (Supplemental Table 7). Low red/processed meat intake was associated with 5% lower risk of stroke mortality than high intake among former smokers (HR: 0.95; 95% CI: 0.91, 0.99).

In the sensitivity analysis among long-term nonsmokers, results across BMI strata showed notable differences from the main analyses (Supplemental Table 8). Diets including white meat-only or low red/processed meat intake were no longer significantly associated with lower risk of all-cause mortality among participants with healthy weight or overweight, respectively. Among those with obesity, a vegetarian diet became associated with 20% lower risk of all-cause mortality than high red/processed meat intake (HR: 0.80; 95% CI: 0.68, 0.95). Additionally, several associations in the age-stratified analyses became nonsignificant.

In the post hoc exploratory analysis with mixed diets (red and white meat) as the reference group, findings for white meat-only and no meat/vegetarian diets were similar to the main analysis, and red meat-only was associated with a 7% to 11% higher risk across mortality outcomes (Supplemental Table 9).

## Discussion

This study of the CPS-II baseline cohort aimed to identify whether diets excluding red/processed meat would confer any benefit beyond limiting intake of these meats for risk of mortality from all causes, heart disease, cancers, and stroke. When all diet categories were compared to high intake of red/processed meat (≥6 d/wk), associations with all-cause and cause-specific mortality risk were generally lower with reduced intakes of red/processed meat, especially with low intake (<4 d/wk), for several subgroups. These included both males and females, individuals with healthy weight or overweight, former or ever-smokers, age groups ≥50 y, and both attained ages (<80 y, ≥80 y). Together, these findings support existing evidence that dietary patterns relatively lower in red and processed meats are associated with positive health outcomes [7]. In partial support of our hypothesis that excluding red/processed meat from the diet could further lower mortality risk, a white meat-only diet was associated with the lowest risk of all-cause mortality among males and individuals with a healthy weight. Both white meat-only and vegetarian diets were associated with the lowest all-cause mortality risk in the 50 to 64 y age group. Among participants reaching age ≥80 y, a white meat-only diet was associated with the lowest risk of both all-cause and cancer mortality. In participants with obesity, only a vegetarian diet was significantly associated with lower mortality risk. Specifically, in sensitivity analyses, all-cause mortality risk was 20% lower in those with obesity who were otherwise healthy at baseline or who were long-term nonsmokers. Although these findings should be interpreted with caution because of generally small effects and low representation of these diets in CPS-II, they indicate that completely excluding red/processed meats may provide additional benefits beyond lowering intake. More studies are needed to further investigate and understand the effects of these dietary choices.

Contrary to our hypothesis, most associations in the vegetarian group were not significant, though this may be due to the low proportion of participants reporting no meat intake in this study. In 2 sensitivity analyses (healthy cohort and excluding the first 2 y of follow-up), a vegetarian diet was associated with a significantly higher risk of all-cause mortality among former smokers. This association may have been driven mostly by former smokers with fewer years since quitting, as the analysis in never-smokers and former smokers of ≥20 y showed mostly null associations for vegetarian diet and all-cause mortality. A vegetarian diet was also associated with a higher risk of stroke mortality among healthy males. However, because few stroke deaths were represented in this subgroup, these findings lack generalizability and should be interpreted with caution. The largely null findings among vegetarians may be partially driven by diet quality, which could not be estimated from the available data. Lower quality vegetarian diets, such as those high in refined grains, added sugars, and sodium-rich foods, or low in essential nutrients like calcium and vitamin B12, have been associated with a higher risk of all-cause mortality and ischemic stroke [32]. Vitamin B12, in particular, aids in neurocognitive health [33], and vitamin B12 supplementation has been associated with lower stroke risk in some populations [34]. A sub-study in the European Prospective Investigation into Cancer and Nutrition-Oxford (EPIC-Oxford) cohort found 7% of male vegetarians were deficient in vitamin B12 compared with <1% of meat-eaters [35]; however, it is not clear whether this translates to a higher risk of stroke mortality among vegetarians. In the Adventist Health Study-2, being vegetarian was associated with higher stroke mortality risk than being nonvegetarian at age 85 [19]; however, a large United Kingdom (UK) Biobank study found no significant difference in stroke mortality risk between vegetarians and meat-eaters [36]. Findings remain inconclusive in our study, where the association between a vegetarian diet and stroke mortality was not significant in the main analysis and had mixed findings in sensitivity analyses. Including strong measures of diet quality in future studies will help elucidate these diet-mortality associations. This is especially important in the context of plant-based dietary patterns, which are increasingly followed in our evolving nutrition landscape with motivations ranging from health to financial, ethical, and environmental impacts [37].

It is difficult to identify comparable studies that have examined diets excluding all red and processed meats while including any white meats (poultry and fish), but forms of these diets have been examined alongside vegetarian diets. An investigation of vegetarians, pesco-vegetarians (eating fish but no other meat), and semi-vegetarians (eating meat up to once a week) in the Australian 45 and Up Study cohort found no associations with all-cause mortality when compared with nonvegetarians over a mean follow-up of 6.1 y; however, no stratification was performed in this analysis [17]. After a similar follow-up time in the Adventist Health Study-2, and also compared with nonvegetarians, males who were vegan, lacto-ovo, or pesco-vegetarians, but not semi-vegetarians, showed 14% to 42% lower risk of all-cause and cardiovascular disease mortality, whereas vegan males and pesco-vegetarian females showed 49% to 55% lower risk of ischemic heart disease mortality [38]. Combining all vegetarians (including pesco-vegetarians and semi-vegetarians), associations with all-cause and cardiovascular disease mortality risk were significantly lower among males but not females. In the large NIH-AARP (formerly known as the American Association of Retired Persons) Diet and Health Study, total white meat intake was inversely associated with mortality from all causes (8%–10% lower risk across white meat quintiles), cancers (9%–16% lower risk), and all other causes (10%–14% lower risk) except cardiovascular diseases and injuries or sudden deaths [1]. Stratification by smoking status identified different associations for processed meat intake and cancer mortality among former/current smokers and never-smokers. These nuanced findings underscore the importance and necessity of examining subgroups when investigating diet/mortality associations. However, even with >988,000 participants in the present study, several subgroup analyses showed low representation of white meat-only and vegetarian diets. Additional population-based studies targeting these diets are needed to better understand their impact on mortality. Studies examining these diets in relation to mortality from specific cancers are also needed, as cancer includes a heterogeneous group of diseases with different etiologies and diet–cancer relationships. A large UK Biobank study examining 19 site-specific incident cancers found 13% lower risk for overall cancers among vegans/vegetarians, 7% lower risk among pescatarians, and no association among fish-poultry eaters compared to meat-eaters (fish, poultry, and red meat) [39]. Few specific sites were associated with significantly lower risk: colorectal (especially colon and proximal colon) and prostate cancer among vegetarians, and melanoma among pescatarians. In the present study, colon and prostate cancers accounted for just 9% and 7% of total cancer deaths, so they could not be examined separately. Yet, colorectal cancer remains a leading cause of cancer death in the United States, and 54% of cases are attributable to potentially modifiable risk factors, including high consumption of red or processed meats [40]. Whether diets excluding these meats may improve colorectal cancer mortality risk remains unclear and requires further examination.

### Strengths and limitations

A major strength of this study is the large sample size of the CPS-II cohort, which represents all parts of the United States and includes a mortality follow-up of over 38.5 y. Cohorts with this length of follow-up and baseline dietary data are unique, as the first United States dietary guidelines were only released in 1980 [7] and the first food frequency questionnaire was published in 1986 [41]. Therefore, CPS-II’s 1982 baseline measurement of dietary intake, including several types of meat, along with demographic, lifestyle, and health factors, facilitated this analysis of diet and mortality risk while accounting for major confounders. After excluding participants with missing data for BMI and smoking status—major risk factors for mortality—we retained as many people as possible to ensure adequate representation of vegetarians. Instead of excluding participants with missing covariate data, we created separate indicator categories; however this approach may have contributed to less precise results, especially for the white-meat-only and vegetarian groups. These groups were much smaller than the red/processed meat groups and showed much wider CI throughout our analyses. Despite having 3,653 vegetarians in our study, which is similar to numbers from many other large cohort studies [20], vegetarians comprised just 0.4% of our analytic sample. We acknowledge that this limited sample may have constrained our ability to detect meaningful differences in vegetarians compared with others, and we interpret the results with caution. Additionally, in the full cohort of nearly 1.2 million participants, few reported consuming only fish (*n* = 516) or chicken (*n* = 223), which limited our ability to examine these meats separately. The disproportionate number of non-Hispanic White participants in the study relative to other races and ethnicities also limits the generalizability of our findings to more diverse populations.

The CPS-II mortality cohort included only a single baseline survey, so any changes in behavior, including diet and health, were not captured over the lengthy follow-up period but may have influenced mortality risk. For instance, updated data on cancer screening and treatments are important considerations for cancer mortality analyses that were not available in this study. In addition, residual confounding cannot be ruled out. The questionnaire did not indicate a timeframe for dietary reporting (e.g., the past month or year), and the lack of dietary detail in the questionnaire may have led to some miscategorization when assigning participants to diet categories in our study. This would mostly impact the white meat-only and vegetarian groups, as participants who failed to report their red/processed meat intake or white meat intake might have been incorrectly assigned to these groups. Furthermore, from the available dietary data, we could not capture serving sizes, estimate total energy intake, or adequately adjust models for total fruit, vegetable, or fiber intake, which are all important diet quality considerations for mortality. This may have further limited our ability to detect effects within diet categories, where high-quality dietary patterns are expected to be more strongly associated with lower risk of all-cause, cardiovascular, and cancer mortality [42]. However, adjusting for limited dietary factors in our sensitivity models did not appreciably change effects. Future research focused on overall dietary alignment with healthy vegetarian dietary patterns can help further clarify these associations. Finally, we acknowledge that competing risks from other diseases may influence the interpretation of our mortality analyses (e.g., non-cancer deaths on cancer mortality). Despite these limitations, findings from this study supported current evidence of the dose–response relationship with mortality risk across reduced levels of red/processed meat intake, and they suggested the potential for additional benefits from fully excluding red/processed meat from the diet.

In conclusion, based on findings from the CPS-II cohort, limiting intake of red and processed meats to <6 d/wk and <4 d/wk was associated with a small but significantly lower risk of mortality from all causes, heart diseases, and cancers among both sexes, different age groups, and individuals without obesity. Excluding all red and processed meats from the diet may further lower mortality risk in some groups, but additional evidence on white meat-only and vegetarian diets in studies targeting these populations and including measures of diet quality is needed to confirm these findings.

## Author contributions

KCC, JS, TJH, AVP, CYU, and MMS-W designed the research; KCC conducted the research, analyzed the data, wrote the paper, and had primary responsibility for the final content. All authors have read and approved the final manuscript.

## Data availability

Data are available from the ACS by following the ACS data access procedures (https://www.cancer.org/research/population-science/research-collaboration.html) for researchers who meet the criteria for access to confidential data. E-mail cohort.data@cancer.org to inquire about access.

## Declaration of Generative AI and AI-assisted technologies in the writing process

The authors declare that no generative AI or AI-assisted technologies were used in the writing of this manuscript.

## Funding

The ACS funds the creation, maintenance, and updating of the Cancer Prevention Study-II cohort. Efforts for KCC on this study were supported through funding from the Woodruff Foundation. The authors assume full responsibility for all analyses and interpretation of results. The views expressed here are those of the authors and do not necessarily represent the ACS or the ACS-Cancer Action Network.

## Conflict of interest

The authors report no conflicts of interest.
