# Supplementary material for: Associations between White Meat-Only and Vegetarian Diets with Mortality from All Causes, Heart Diseases, Cancers, and Stroke in the American Cancer Society’s Cancer Prevention Study-II
Source: Curr Dev Nutr. 2026 Jul 17;10(8):109438. doi: 10.1016/j.cdnut.2026.109438 (PMC13475420; doi:10.1016/j.cdnut.2026.109438)
Supplement: Supplementary file 1 [file mmc1.docx]

**Title:** Associations between white meat-only and vegetarian diets with mortality from all causes, heart diseases, cancers, and stroke in the American Cancer Society’s Cancer Prevention Study-II

**Authors:** Kelly C. Cara, Joan Sabaté, Terryl J. Hartman, Alpa V. Patel, Caroline Y. Um, Marissa M. Shams-White

**Supplemental Materials**

**Table of Contents**

[Supplemental Table 1. Risk of all-cause and cause-specific mortality by diet categories in the full analytic cohort (*n =* 988,378) with results across statistical models^1^	2](#_Toc236236885)

[Supplemental Table 2. Risk of all-cause and cause-specific^1^ mortality by diet categories and stratification by subgroups^2^ with results from fully adjusted models^3^ in the full analytic cohort (*n =* 988,378)	3](#_Toc236236886)

[Supplemental Table 3. Risk of all-cause and cause-specific mortality by diet categories with results from fully adjusted models^1^ in the main analysis and sensitivity analyses	6](#_Toc236236887)

[Supplemental Table 4. Risk of all-cause mortality by diet categories and stratification by subgroups^1^ with results from fully adjusted models^2^ in the main analysis and sensitivity analyses	8](#_Toc236236888)

[Supplemental Table 5. Risk of heart disease mortality by diet categories and stratification by subgroups^1^ with results from fully adjusted models^2^ in the main analysis and sensitivity analyses	11](#_Toc236236889)

[Supplemental Table 6. Risk of cancer mortality by diet categories and stratification by subgroups^1^ with results from fully adjusted models^2^ in the main analysis and sensitivity analyses	14](#_Toc236236890)

[Supplemental Table 7. Risk of stroke mortality by diet categories and stratification by subgroups^1^ with results from fully adjusted models^2^ in the main analysis and sensitivity analyses	17](#_Toc236236891)

[Supplemental Table 8. Risk of all-cause mortality by diet categories overall and stratified by subgroups^1^ with results across statistical models^2^ in the sensitivity analyses including only long-term non-smokers^3^	19](#_Toc236236892)

[Supplemental Table 9. Risk of all-cause and cause-specific mortality in the full analytic cohort (*n =* 988,378) with results by meat type and across statistical models in the post-hoc exploratory analysis^1^	21](#_Toc236236893)

Supplemental Table 1. Risk of all-cause and cause-specific mortality by diet categories in the full analytic cohort (*n =* 988,378) with results across statistical models^1^

| **Cause of mortality** | **Deaths/person-years** | **Model 1**  Age | **Model 2**  Demographics | **Model 3**  Lifestyle | **Model 4**  Health | **Model 5**  BMI |
| --- | --- | --- | --- | --- | --- | --- |
| **All causes** |  |  |  |  |  |  |
| Red/pr meat - High | 226,783/9,531,167 | *Reference* | *Reference* | *Reference* | *Reference* | *Reference* |
| Red/pr meat - Moderate | 190,140/7,929,461 | **0.93^‡^** (0.92, 0.93) | **0.97^‡^** (0.96, 0.97) | **0.98^‡^** (0.97, 0.98) | **0.98^‡^** (0.98, 0.99) | **0.99^‡^** (0.98, 0.99) |
| Red/pr meat - Low | 201,402/7,597,227 | **0.91^‡^** (0.90, 0.91) | **0.95^‡^** (0.94, 0.95) | **0.96^‡^** (0.96, 0.97) | **0.97^‡^** (0.96, 0.97) | **0.98^‡^** (0.97, 0.98) |
| White meat only | 5,495/206,327 | **0.86^‡^** (0.83, 0.88) | **0.91^‡^** (0.89, 0.93) | **0.94^‡^** (0.92, 0.97) | **0.94^‡^** (0.91, 0.96) | **0.96^†^** (0.93, 0.98) |
| No meat/vegetarian | 2,197/87,110 | **0.83^‡^** (0.80, 0.87) | **0.88^‡^** (0.84, 0.92) | **0.93^†^** (0.90, 0.97) | 0.96 (0.92, 1.00) | 0.97 (0.93, 1.01) |
| **Diseases of the heart** |  |  |  |  |  |  |
| Red/pr meat - High | 62,868/9,531,167 | *Reference* | *Reference* | *Reference* | *Reference* | *Reference* |
| Red/pr meat - Moderate | 53,136/7,929,461 | **0.92^‡^** (0.91, 0.93) | **0.97^‡^** (0.96, 0.98) | **0.98^‡^** (0.97, 0.99) | **0.97^‡^** (0.96, 0.98) | **0.98^†^** (0.97, 1.00) |
| Red/pr meat - Low | 60,033/7,597,227 | **0.92^‡^** (0.90, 0.93) | **0.98^‡^** (0.97, 0.99) | **0.98^†^** (0.97, 1.00) | **0.96^‡^** (0.95, 0.98) | **0.99*** (0.97, 1.00) |
| White meat only | 1,754/206,327 | **0.90^‡^** (0.86, 0.95) | 0.98 (0.94, 1.03) | 1.00 (0.95, 1.04) | **0.94*** (0.90, 0.99) | 0.99 (0.94, 1.04) |
| No meat/vegetarian | 678/87,110 | **0.84^‡^** (0.78, 0.90) | **0.91*** (0.84, 0.98) | 0.93 (0.86, 1.00) | 0.96 (0.89, 1.03) | 0.99 (0.92, 1.07) |
| **Cancers** |  |  |  |  |  |  |
| Red/pr meat - High | 60,799/9,531,167 | *Reference* | *Reference* | *Reference* | *Reference* | *Reference* |
| Red/pr meat - Moderate | 49,811/7,929,461 | **0.94^‡^** (0.92, 0.95) | **0.97^‡^** (0.96, 0.99) | **0.99*** (0.97, 1.00) | 0.99 (0.98, 1.00) | 0.99 (0.98, 1.00) |
| Red/pr meat - Low | 49,590/7,597,227 | **0.89^‡^** (0.88, 0.91) | **0.94^‡^** (0.93, 0.95) | **0.97^‡^** (0.96, 0.98) | **0.97^‡^** (0.96, 0.98) | **0.98^‡^** (0.97, 0.99) |
| White meat only | 1,280/206,327 | **0.83^‡^** (0.78, 0.87) | **0.88^‡^** (0.84, 0.93) | 0.95 (0.90, 1.01) | 0.96 (0.91, 1.01) | 0.97 (0.92, 1.03) |
| No meat/vegetarian | 512/87,110 | **0.80^‡^** (0.74, 0.88) | **0.86^‡^** (0.78, 0.93) | 0.98 (0.90, 1.07) | 0.98 (0.90, 1.07) | 0.99 (0.91, 1.08) |
| **Stroke** |  |  |  |  |  |  |
| Red/pr meat - High | 15,012/9,531,167 | *Reference* | *Reference* | *Reference* | *Reference* | *Reference* |
| Red/pr meat - Moderate | 13,421/7,929,461 | **0.96^‡^** (0.94, 0.98) | 0.99 (0.96, 1.01) | 0.99 (0.97, 1.02) | 1.00 (0.97, 1.02) | 1.00 (0.98, 1.02) |
| Red/pr meat - Low | 14,697/7,597,227 | **0.92^‡^** (0.90, 0.94) | **0.95^‡^** (0.93, 0.97) | **0.96^‡^** (0.94, 0.98) | **0.96^†^** (0.94, 0.99) | **0.96^†^** (0.94, 0.99) |
| White meat only | 420/206,327 | **0.88*** (0.80, 0.97) | **0.91*** (0.82, 1.00) | 0.92 (0.84, 1.02) | 0.93 (0.84, 1.02) | 0.93 (0.84, 1.03) |
| No meat/vegetarian | 200/87,110 | 1.01 (0.88, 1.16) | 1.02 (0.89, 1.17) | 1.05 (0.91, 1.20) | 1.09 (0.94, 1.25) | 1.09 (0.94, 1.25) |

HR (95% CI) from Cox proportional hazards models with statistically significant results in bold: ^*^ *P* < 0.05, ^†^ *P* < 0.01, ^‡^ *P* < 0.001; pr, processed

^1^ Statistical models: **Model 1** = mortality outcome ~ exposure + strata (age); **Model 2** = Model 1 + demographic factors demographics (sex, race, education level, marital status, U.S. Census Bureau region); **Model 3** = Model 2 + lifestyle factors (12-level smoking status, alcohol intake, physical activity, sleep); **Model 4** = Model 3 + health history (Unique covariates for each mortality outcome were as follows. *All causes*: number of comorbidities, high blood pressure; *Heart diseases:* history of heart disease, high blood pressure, menopause status, hormone replacement therapy; *Cancers*: history of cancer, family history of cancer, history of diabetes, menopause status, hormone replacement therapy; *Stroke*: history of stroke, high blood pressure, history of diabetes, menopause status, hormone replacement therapy); **Model 5 (fully adjusted)** = Model 4 + 6-level BMI

Supplemental Table 2. Risk of all-cause and cause-specific^1^ mortality by diet categories and stratification by subgroups^2^ with results from fully adjusted models^3^ in the full analytic cohort (*n =* 988,378)

|  | **All-cause mortality** | |  | **Heart disease mortality** | |  | **Cancer mortality** | |
| --- | --- | --- | --- | --- | --- | --- | --- | --- |
| **Participants analyzed** | **Deaths/person-years** | **Fully adjusted model** |  | **Deaths/person-years** | **Fully adjusted model** |  | **Deaths/person-years** | **Fully adjusted model** |
| **SEX** |  |  |  | No significant interactions^2^ | |  |  |  |
| **Males** | ***n =* 442,437** |  |  |  |  |  | ***n =* 442,437** |  |
| Red/pr meat - High | 123,154/4,418,143 | *Reference* |  |  |  |  | 35,131/4,418,143 | *Reference* |
| Red/pr meat - Moderate | 86,955/3,076,934 | **0.99^*^** (0.98, 1.00) |  |  |  |  | 24,360/3,076,934 | 0.98 (0.97, 1.00) |
| Red/pr meat - Low | 82,572/2,713,499 | **0.98^‡^** (0.97, 0.99) |  |  |  |  | 22,243/2,713,499 | **0.98^*^** (0.96, 1.00) |
| White meat only | 1,867/63,439 | **0.93^†^** (0.89, 0.98) |  |  |  |  | 476/63,439 | 0.96 (0.87, 1.05) |
| No meat/vegetarian | 812/30,311 | 0.95 (0.88, 1.01) |  |  |  |  | 209/30,311 | 0.95 (0.83, 1.09) |
| **Females** | ***n =* 545,941** |  |  |  |  |  | ***n =* 545,941** |  |
| Red/pr meat - High | 103,629/5,113,024 | *Reference* |  |  |  |  | 25,668/5,113,024 | *Reference* |
| Red/pr meat - Moderate | 103,185/4,852,527 | **0.99^*^** (0.98, 1.00) |  |  |  |  | 25,451/4,852,527 | 1.00 (0.98, 1.02) |
| Red/pr meat - Low | 118,830/4,883,727 | **0.98^‡^** (0.97, 0.99) |  |  |  |  | 27,347/4,883,727 | **0.98^*^** (0.97, 1.00) |
| White meat only | 3,628/142,888 | 0.98 (0.95, 1.01) |  |  |  |  | 804/142,888 | 0.99 (0.92, 1.06) |
| No meat/vegetarian | 1,385/56,798 | 0.99 (0.94, 1.05) |  |  |  |  | 303/56,798 | 1.02 (0.91, 1.14) |
| **BMI CATEGORY** |  |  |  |  |  |  | No significant interactions^2^ | |
| **Healthy weight** | ***n =* 509,633** |  |  | ***n =* 509,633** |  |  |  |  |
| Red/pr meat - High | 100,512/4,692,919 | *Reference* |  | 24,474/4,692,919 | *Reference* |  |  |  |
| Red/pr meat - Moderate | 93,455/4,293,463 | **0.98^‡^** (0.97, 0.99) |  | 23,183/4,293,463 | **0.97^‡^** (0.95, 0.99) |  |  |  |
| Red/pr meat - Low | 103,782/4,273,462 | **0.97^‡^** (0.96, 0.98) |  | 27,907/4,273,462 | **0.97^‡^** (0.95, 0.99) |  |  |  |
| White meat only | 3,187/133,098 | **0.95^†^** (0.91, 0.98) |  | 933/133,098 | 0.96 (0.90, 1.03) |  |  |  |
| No meat/vegetarian | 1,295/54,727 | 1.01 (0.95, 1.06) |  | 375/54,727 | 1.03 (0.93, 1.14) |  |  |  |
| **Overweight** | ***n =* 369,608** |  |  | ***n =* 369,608** |  |  |  |  |
| Red/pr meat - High | 93,246/3,674,405 | *Reference* |  | 27,383/3,674,405 | *Reference* |  |  |  |
| Red/pr meat - Moderate | 73,569/2,838,602 | 0.99 (0.98, 1.00) |  | 22,328/2,838,602 | 1.00 (0.98, 1.02) |  |  |  |
| Red/pr meat - Low | 74,814/2,616,677 | **0.99^*^** (0.98, 1.00) |  | 24,169/2,616,677 | 1.01 (0.99, 1.03) |  |  |  |
| White meat only | 1,785/58,516 | 0.97 (0.93, 1.02) |  | 615/58,516 | 1.00 (0.92, 1.08) |  |  |  |
| No meat/vegetarian | 702/25,259 | 0.94 (0.88, 1.02) |  | 230/25,259 | 0.96 (0.84, 1.09) |  |  |  |
| **Obesity** | ***n =* 109,137** |  |  | ***n =* 109,137** |  |  |  |  |
| Red/pr meat - High | 33,025/1,163,842 | *Reference* |  | 11,011/1,163,842 | *Reference* |  |  |  |
| Red/pr meat - Moderate | 23,116/797,396 | 1.01 (0.99, 1.02) |  | 7,625/797,396 | 0.99 (0.96, 1.01) |  |  |  |
| Red/pr meat - Low | 22,806/707,088 | 1.00 (0.98, 1.01) |  | 7,957/707,088 | 1.00 (0.97, 1.03) |  |  |  |
| White meat only | 523/14,713 | 0.99 (0.91, 1.08) |  | 206/14,713 | 1.08 (0.94, 1.24) |  |  |  |
| No meat/vegetarian | 200/7,124 | 0.87 (0.76, 1.00) |  | 73/7,124 | 0.90 (0.72, 1.14) |  |  |  |
| **SMOKING STATUS** |  |  |  |  |  |  |  |  |
| **Never smokers** | ***n =* 417,041** |  |  | ***n =* 417,041** |  |  | ***n =* 417,041** |  |
| Red/pr meat - High | 80,783/4,128,445 | *Reference* |  | 22,759/4,128,445 | *Reference* |  | 18,175/4,128,445 | *Reference* |
| Red/pr meat - Moderate | 72,548/3,551,808 | 0.99 (0.98, 1.00) |  | 20,796/3,551,808 | 0.99 (0.97, 1.01) |  | 16,108/3,551,808 | 1.00 (0.98, 1.02) |
| Red/pr meat - Low | 82,404/3,471,985 | 0.99 (0.98, 1.00) |  | 25,384/3,471,985 | 1.00 (0.98, 1.02) |  | 17,066/3,471,985 | 0.99 (0.97, 1.02) |
| White meat only | 2,539/100,046 | 0.98 (0.94, 1.02) |  | 828/100,046 | 0.99 (0.93, 1.07) |  | 540/100,046 | 1.05 (0.97, 1.15) |
| No meat/vegetarian | 1,301/56,624 | 0.97 (0.92, 1.03) |  | 407/56,624 | 0.98 (0.89, 1.09) |  | 271/56,624 | 0.98 (0.87, 1.11) |
| **Former smokers** | ***n =* 293,766** |  |  | ***n =* 293,766** |  |  | ***n =* 293,766** |  |
| Red/pr meat - High | 67,031/2,730,612 | *Reference* |  | 19,315/2,730,612 | *Reference* |  | 17,952/2,730,612 | *Reference* |
| Red/pr meat - Moderate | 57,151/2,314,718 | **0.99^*^** (0.98, 1.00) |  | 16,753/2,314,718 | 0.99 (0.97, 1.01) |  | 15,031/2,314,718 | 0.99 (0.97, 1.01) |
| Red/pr meat - Low | 60,140/2,253,285 | **0.96^‡^** (0.95, 0.98) |  | 18,345/2,253,285 | **0.97^†^** (0.95, 0.99) |  | 15,268/2,253,285 | **0.97^†^** (0.95, 0.99) |
| White meat only | 1,684/67,737 | 0.96 (0.91, 1.01) |  | 561/67,737 | 1.03 (0.94, 1.12) |  | 405/67,737 | 0.94 (0.85, 1.04) |
| No meat/vegetarian | 483/18,783 | 1.08 (0.99, 1.19) |  | 142/18,783 | 1.10 (0.93, 1.30) |  | 127/18,783 | 1.13 (0.95, 1.35) |
| **Ever smokers** | ***n =* 59,982** |  |  | ***n =* 59,982** |  |  | ***n =* 59,982** |  |
| Red/pr meat - High | 14,779/538,229 | *Reference* |  | 4,649/538,229 | *Reference* |  | 3,826/538,229 | *Reference* |
| Red/pr meat - Moderate | 11,292/410,774 | 0.98 (0.95, 1.00) |  | 3,503/410,774 | **0.95^*^** (0.91, 0.99) |  | 2,973/410,774 | 1.00 (0.95, 1.05) |
| Red/pr meat - Low | 12,675/421,313 | **0.96^†^** (0.94, 0.99) |  | 4,311/421,313 | 0.98 (0.94, 1.02) |  | 3,098/421,313 | **0.95^*^** (0.91, 1.00) |
| White meat only | 364/11,119 | 0.95 (0.85, 1.05) |  | 119/11,119 | 0.90 (0.75, 1.08) |  | 82/11,119 | 0.91 (0.73, 1.13) |
| No meat/vegetarian | 127/3,320 | 1.00 (0.84, 1.19) |  | 49/3,320 | 1.06 (0.80, 1.41) |  | 29/3,320 | 1.00 (0.69, 1.44) |
| **Current smokers** | ***n =* 217,589** |  |  | ***n =* 217,589** |  |  | ***n =* 217,589** |  |
| Red/pr meat - High | 64,190/2,133,880 | *Reference* |  | 16,145/2,133,880 | *Reference* |  | 20,846/2,133,880 | *Reference* |
| Red/pr meat - Moderate | 49,149/1,652,160 | 1.00 (0.98, 1.01) |  | 12,084/1,652,160 | 0.99 (0.96, 1.01) |  | 15,699/1,652,160 | 1.00 (0.98, 1.02) |
| Red/pr meat - Low | 46,183/1,450,644 | 0.99 (0.97, 1.00) |  | 11,993/1,450,644 | 0.99 (0.96, 1.01) |  | 14,158/1,450,644 | 1.00 (0.97, 1.02) |
| White meat only | 908/27,424 | 0.96 (0.88, 1.03) |  | 246/27,424 | 0.96 (0.82, 1.12) |  | 253/27,424 | 0.90 (0.77, 1.05) |
| No meat/vegetarian | 286/8,383 | 0.93 (0.81, 1.08) |  | 80/8,383 | 0.87 (0.65, 1.15) |  | 85/8,383 | 1.03 (0.80, 1.33) |
| **AGE GROUP** |  |  |  |  |  |  |  |  |
| **Less than 50 y** | ***n =* 257,856** |  |  | ***n =* 257,856** |  |  | ***n =* 257,856** |  |
| Red/pr meat - High | 43,787/3,753,895 | *Reference* |  | 9,437/3,753,895 | *Reference* |  | 14,457/3,753,895 | *Reference* |
| Red/pr meat - Moderate | 30,157/2,785,951 | 1.00 (0.98, 1.01) |  | 6,247/2,785,951 | 1.00 (0.97, 1.03) |  | 10,243/2,785,951 | 1.00 (0.98, 1.03) |
| Red/pr meat - Low | 23,298/2,144,495 | 0.99 (0.98, 1.01) |  | 5,161/2,144,495 | **1.05^†^** (1.02, 1.09) |  | 7,548/2,144,495 | 0.97 (0.95, 1.00) |
| White meat only | 525/56,641 | 0.97 (0.89, 1.06) |  | 115/56,641 | 1.05 (0.87, 1.26) |  | 195/56,641 | 1.10 (0.96, 1.27) |
| No meat/vegetarian | 266/28,825 | 1.09 (0.96, 1.23) |  | 46/28,825 | 0.94 (0.70, 1.26) |  | 98/28,825 | 1.22 (1.00, 1.49) |
| **50 to 64 y** | ***n =* 514,981** |  |  | ***n =* 514,981** |  |  | ***n =* 514,981** |  |
| Red/pr meat - High | 135,375/4,887,174 | *Reference* |  | 36,556/4,887,174 | *Reference* |  | 35,878/4,887,174 | *Reference* |
| Red/pr meat - Moderate | 113,914/4,240,937 | **0.99^†^** (0.98, 1.00) |  | 30,337/4,240,937 | **0.98^*^** (0.97, 1.00) |  | 29,622/4,240,937 | 0.99 (0.97, 1.00) |
| Red/pr meat - Low | 113,628/4,188,521 | **0.97^‡^** (0.97, 0.98) |  | 31,403/4,188,521 | **0.98^*^** (0.97, 1.00) |  | 28,718/4,188,521 | **0.98^†^** (0.96, 0.99) |
| White meat only | 2,791/108,441 | **0.94^‡^** (0.90, 0.97) |  | 779/108,441 | 0.94 (0.87, 1.01) |  | 689/108,441 | 0.96 (0.89, 1.03) |
| No meat/vegetarian | 993/40,492 | **0.94^*^** (0.88, 1.00) |  | 271/40,492 | 0.94 (0.84, 1.06) |  | 235/40,492 | 0.93 (0.82, 1.06) |
| **65 y or more** | ***n =* 215,541** |  |  | ***n =* 215,541** |  |  | ***n =* 215,541** |  |
| Red/pr meat - High | 47,621/890,097 | *Reference* |  | 16,875/890,097 | *Reference* |  | 10,464/890,097 | *Reference* |
| Red/pr meat - Moderate | 46,069/902,573 | **0.98^†^** (0.97, 0.99) |  | 16,552/902,573 | 0.98 (0.96, 1.00) |  | 9,946/902,573 | 0.99 (0.96, 1.02) |
| Red/pr meat - Low | 64,476/1,264,210 | **0.97^‡^** (0.96, 0.98) |  | 23,469/1,264,210 | **0.97^†^** (0.95, 0.99) |  | 13,324/1,264,210 | 0.98 (0.95, 1.00) |
| White meat only | 2,179/41,245 | 0.99 (0.95, 1.03) |  | 860/41,245 | 1.03 (0.96, 1.10) |  | 396/41,245 | 0.94 (0.85, 1.04) |
| No meat/vegetarian | 938/17,793 | 0.98 (0.92, 1.05) |  | 361/17,793 | 1.03 (0.92, 1.14) |  | 179/17,793 | 0.96 (0.83, 1.12) |
| **ATTAINED AGE** |  |  |  |  |  |  |  |  |
| **Less than 80 y** | ***n =* 972,717** |  |  | ***n =* 972,717** |  |  | ***n =* 972,717** |  |
| Red/pr meat - High | 106,100/7,818,882 | *Reference* |  | 28,439/7,818,882 | *Reference* |  | 38,920/7,818,882 | *Reference* |
| Red/pr meat - Moderate | 81,658/6,352,383 | **0.99^*^** (0.98, 1.00) |  | 22,004/6,352,383 | 0.99 (0.98, 1.01) |  | 30,410/6,352,383 | **0.98^*^** (0.97, 1.00) |
| Red/pr meat - Low | 80,077/5,853,832 | **0.98^‡^** (0.97, 0.99) |  | 22,952/5,853,832 | 0.99 (0.97, 1.01) |  | 28,809/5,853,832 | **0.97^‡^** (0.96, 0.99) |
| White meat only | 2,102/155,546 | 1.00 (0.96, 1.05) |  | 628/155,546 | 1.01 (0.93, 1.09) |  | 752/155,546 | 1.02 (0.95, 1.10) |
| No meat/vegetarian | 809/66,214 | 1.02 (0.95, 1.10) |  | 224/66,214 | 1.01 (0.89, 1.15) |  | 292/66,214 | 1.03 (0.92, 1.16) |
| **80 y or more** | ***n =* 668,289** |  |  | ***n =* 668,289** |  |  | ***n =* 668,289** |  |
| Red/pr meat - High | 120,683/1,712,284 | *Reference* |  | 34,429/1,712,284 | *Reference* |  | 21,879/1,712,284 | *Reference* |
| Red/pr meat - Moderate | 108,482/1,577,077 | **0.99^*^** (0.98, 1.00) |  | 31,132/1,577,077 | **0.98^†^** (0.96, 0.99) |  | 19,401/1,577,077 | 1.00 (0.99, 1.02) |
| Red/pr meat - Low | 121,325/1,743,394 | **0.98^‡^** (0.97, 0.98) |  | 37,081/1,743,394 | **0.98^†^** (0.97, 1.00) |  | 20,781/1,743,394 | 0.99 (0.97, 1.01) |
| White meat only | 3,393/50,781 | **0.94^‡^** (0.91, 0.97) |  | 1,126/50,781 | 0.98 (0.92, 1.04) |  | 528/50,781 | **0.91^*^** (0.83, 0.99) |
| No meat/vegetarian | 1,388/20,896 | 0.95 (0.90, 1.00) |  | 454/20,896 | 0.98 (0.90, 1.08) |  | 220/20,896 | 0.94 (0.82, 1.07) |

HR (95% CI) from Cox proportional hazards models with statistically significant results in bold: ^*^ *P* < 0.05, ^†^ *P* < 0.01, ^‡^ *P* < 0.001; pr, processed

^1^ The effect of diet on stroke mortality was not modified by any factor tested, so stroke mortality is not presented.

^2^ Subgroups are based on formal interaction tests showing significant effect modification (otherwise, cells are left blank); The interactions tested were each added separately to Model 5 as follows: diet^*^sex, diet^*^simple BMI category, diet^*^simple smoking status, diet^*^age group, diet^*^attained age.

^3^ Model 5 (fully adjusted) = mortality outcome ~ exposure + strata (age) + demographic factors demographics (sex [not adjusted in sex strata], race, education level, marital status, U.S. Census Bureau region) + lifestyle factors (12-level smoking status [not adjusted in smoking strata; replaced with years since quit (*Former* *smokers*) or cigarettes per day with years (*Current smokers*)], alcohol intake, physical activity, sleep) + health history (Unique covariates for each mortality outcome were as follows. *All causes*: number of comorbidities, high blood pressure; *Heart diseases:* history of heart disease, high blood pressure, menopause status, hormone replacement therapy; *Cancers*: history of cancer, family history of cancer, history of diabetes, menopause status, hormone replacement therapy; *Stroke*: history of stroke, high blood pressure, history of diabetes, menopause status, hormone replacement therapy) + 6-level BMI (continuous BMI for BMI strata)

Supplemental Table 3. Risk of all-cause and cause-specific mortality by diet categories with results from fully adjusted models^1^ in the main analysis and sensitivity analyses

|  | **Full analytic cohort**  *n =* 988,378 | | |  | **Healthy cohort^2^**  *n =* 724,311 | |  | **Excludes first 2 y of follow-up**  *n =* 971,703 | |
| --- | --- | --- | --- | --- | --- | --- | --- | --- | --- |
| **Cause of mortality** | **Deaths/person-years** | **Fully adjusted model** | **Fully adjusted  + diet factors^3^** |  | **Deaths/person-years** | **Fully adjusted model** |  | **Deaths/person-years** | **Fully adjusted model** |
| **All causes** |  |  |  |  |  |  |  |  |  |
| Red/pr meat - High | 226,783/9,531,167 | *Reference* | *Reference* |  | 155,908/7,534,602 | *Reference* |  | 221,231/9,525,123 | *Reference* |
| Red/pr meat - Mod | 190,140/7,929,461 | **0.99^‡^** (0.98, 0.99) | **0.99^‡^** (0.98, 0.99) |  | 131,257/6,296,747 | **0.98^‡^** (0.98, 0.99) |  | 185,505/7,924,407 | **0.99^‡^** (0.98, 0.99) |
| Red/pr meat - Low | 201,402/7,597,227 | **0.98^‡^** (0.97, 0.98) | **0.97^‡^** (0.96, 0.97) |  | 134,094/5,876,429 | **0.97^‡^** (0.97, 0.98) |  | 195,651/7,591,003 | **0.98^‡^** (0.97, 0.98) |
| White meat only | 5,495/206,327 | **0.96^†^** (0.93, 0.98) | **0.95^‡^** (0.92, 0.97) |  | 3,391/151,748 | **0.95^†^** (0.92, 0.98) |  | 5,291/206,121 | **0.95^‡^** (0.93, 0.98) |
| No meat/vegetarian | 2,197/87,110 | 0.97 (0.93, 1.01) | 0.96 (0.93, 1.01) |  | 1,479/67,893 | 1.01 (0.96, 1.06) |  | 2,119/87,035 | 0.97 (0.93, 1.02) |
| **Diseases of the heart** |  |  |  |  |  |  |  |  |  |
| Red/pr meat - High | 62,868/9,531,167 | *Reference* | *Reference* |  | 39,949/7,534,602 | *Reference* |  | 60,821/9,525,123 | *Reference* |
| Red/pr meat - Mod | 53,136/7,929,461 | **0.98^†^** (0.97, 1.00) | **0.98^†^** (0.97, 0.99) |  | 33,645/6,296,747 | **0.98^†^** (0.96, 0.99) |  | 51,391/7,924,407 | **0.98^†^** (0.97, 1.00) |
| Red/pr meat - Low | 60,033/7,597,227 | **0.99^*^** (0.97, 1.00) | **0.98^‡^** (0.97, 0.99) |  | 36,496/5,876,429 | 0.99 (0.97, 1.00) |  | 57,825/7,591,003 | **0.99^*^** (0.97, 1.00) |
| White meat only | 1,754/206,327 | 0.99 (0.94, 1.04) | 0.98 (0.93, 1.03) |  | 978/151,748 | 0.99 (0.93, 1.06) |  | 1,665/206,121 | 0.98 (0.93, 1.03) |
| No meat/vegetarian | 678/87,110 | 0.99 (0.92, 1.07) | 0.99 (0.92, 1.07) |  | 421/67,893 | 1.04 (0.94, 1.14) |  | 640/87,035 | 0.98 (0.91, 1.06) |
| **Cancers** |  |  |  |  |  |  |  |  |  |
| Red/pr meat - High | 60,799/9,531,167 | *Reference* | *Reference* |  | 43,334/7,534,602 | *Reference* |  | 58,704/9,525,123 | *Reference* |
| Red/pr meat - Mod | 49,811/7,929,461 | 0.99 (0.98, 1.00) | 0.99 (0.98, 1.00) |  | 35,339/6,296,747 | 0.99 (0.97, 1.00) |  | 48,031/7,924,407 | 0.99 (0.98, 1.00) |
| Red/pr meat - Low | 49,590/7,597,227 | **0.98^‡^** (0.97, 0.99) | **0.97^‡^** (0.96, 0.98) |  | 34,032/5,876,429 | **0.98^†^** (0.96, 0.99) |  | 47,551/7,591,003 | **0.98^†^** (0.97, 0.99) |
| White meat only | 1,280/206,327 | 0.97 (0.92, 1.03) | 0.96 (0.91, 1.02) |  | 792/151,748 | 0.94 (0.87, 1.01) |  | 1,212/206,121 | 0.97 (0.91, 1.02) |
| No meat/vegetarian | 512/87,110 | 0.99 (0.91, 1.08) | 0.98 (0.90, 1.07) |  | 351/67,893 | 1.02 (0.92, 1.13) |  | 490/87,035 | 1.00 (0.92, 1.09) |
| **Stroke** |  |  |  |  |  |  |  |  |  |
| Red/pr meat - High | 15,012/9,531,167 | *Reference* | *Reference* |  | 10,451/7,534,602 | *Reference* |  | 14,708/9,525,123 | *Reference* |
| Red/pr meat - Mod | 13,421/7,929,461 | 1.00 (0.98, 1.02) | 1.00 (0.97, 1.02) |  | 9,504/6,296,747 | 1.01 (0.98, 1.03) |  | 13,157/7,924,407 | 1.00 (0.98, 1.02) |
| Red/pr meat - Low | 14,697/7,597,227 | **0.96^†^** (0.94, 0.99) | **0.96^‡^** (0.93, 0.98) |  | 10,103/5,876,429 | 0.97 (0.95, 1.00) |  | 14,331/7,591,003 | **0.96^†^** (0.94, 0.99) |
| White meat only | 420/206,327 | 0.93 (0.84, 1.03) | 0.92 (0.83, 1.01) |  | 261/151,748 | 0.91 (0.80, 1.03) |  | 408/206,121 | 0.93 (0.84, 1.03) |
| No meat/vegetarian | 200/87,110 | 1.09 (0.94, 1.25) | 1.07 (0.93, 1.23) |  | 144/67,893 | **1.19^*^** (1.01, 1.41) |  | 194/87,035 | 1.09 (0.94, 1.26) |

HR (95% CI) from Cox proportional hazards models with statistically significant results in bold: ^*^ *P* < 0.05, ^†^ *P* < 0.01, ^‡^ *P* < 0.001; mod, moderate; pr, processed

^1^ Model 5 (fully adjusted) = mortality outcome ~ exposure + strata (age) + demographic factors demographics (sex [not adjusted in sex strata], race, education level, marital status, U.S. Census Bureau region) + lifestyle factors (12-level smoking status [not adjusted in smoking strata; replaced with years since quit (*Former* *smokers*) or cigarettes per day with years (*Current smokers*)], alcohol intake, physical activity, sleep) + health history (Unique covariates for each mortality outcome were as follows, with items in bold excluded from the healthy cohort analyses. *All causes*: **number of comorbidities**, high blood pressure; *Heart diseases:* **history of heart disease**, high blood pressure, menopause status, hormone replacement therapy; *Cancers*: **history of cancer**, family history of cancer, **history of diabetes**, menopause status, hormone replacement therapy; *Stroke*: **history of stroke**, high blood pressure, **history of diabetes**, menopause status, hormone replacement therapy) + 6-level BMI (continuous BMI for BMI strata)

^2^ Exclusions were as follows: History of heart diseases, cancers (except for non-melanoma skin cancers), stroke, chronic respiratory conditions (emphysema, chronic bronchitis, or asthma), diabetes mellitus, chronic kidney diseases, chronic liver diseases

^3^ Additionally adjusted for dietary intake (d/wk) of total vegetables, whole grains, citrus fruits/juices

Supplemental Table 4. Risk of all-cause mortality by diet categories and stratification by subgroups^1^ with results from fully adjusted models^2^ in the main analysis and sensitivity analyses

|  | **Full analytic cohort**  *n =* 988,378 | |  | **Healthy cohort^3^**  *n =* 724,311 | |  | **Excludes first 2 y of follow-up**  *n =* 971,703 | |  |
| --- | --- | --- | --- | --- | --- | --- | --- | --- | --- |
| **Participants analyzed** | **Deaths/person-years** | **Fully adjusted model** |  | **Deaths/person-years** | **Fully adjusted model** |  | **Deaths/person-years** | **Fully adjusted model** | |
| **SEX** |  |  |  |  |  |  |  |  | |
| **Males** | ***n =* 442,437** |  |  | ***n =* 318,434** |  |  | ***n =* 431,892** |  | |
| Red/pr meat - High | 123,154/4,418,143 | *Reference* |  | 83,995/3,504,121 | *Reference* |  | 119,318/4,414,021 | *Reference* | |
| Red/pr meat - Moderate | 86,955/3,076,934 | **0.99^*^** (0.98, 1.00) |  | 58,829/2,443,164 | **0.98^‡^** (0.97, 0.99) |  | 84,078/3,073,817 | **0.99^†^** (0.98, 1.00) | |
| Red/pr meat - Low | 82,572/2,713,499 | **0.98^‡^** (0.97, 0.99) |  | 52,910/2,080,390 | **0.97^‡^** (0.96, 0.98) |  | 79,176/2,709,858 | **0.98^‡^** (0.97, 0.99) | |
| White meat only | 1,867/63,439 | **0.93^†^** (0.89, 0.98) |  | 1,083/44,821 | 0.96 (0.90, 1.02) |  | 1,775/63,346 | **0.93^†^** (0.89, 0.98) | |
| No meat/vegetarian | 812/30,311 | 0.95 (0.88, 1.01) |  | 540/23,905 | 1.01 (0.93, 1.10) |  | 777/30,280 | 0.95 (0.89, 1.02) | |
| **Females** | ***n =* 545,941** |  |  | ***n =* 405,877** |  |  | ***n =* 539,811** |  | |
| Red/pr meat - High | 103,629/5,113,024 | *Reference* |  | 71,913/4,030,481 | *Reference* |  | 101,913/5,111,102 | *Reference* | |
| Red/pr meat - Moderate | 103,185/4,852,527 | **0.99^*^** (0.98, 1.00) |  | 72,428/3,853,583 | **0.99^*^** (0.98, 1.00) |  | 101,427/4,850,590 | **0.99^*^** (0.98, 1.00) | |
| Red/pr meat - Low | 118,830/4,883,727 | **0.98^‡^** (0.97, 0.99) |  | 81,184/3,796,039 | **0.98^‡^** (0.97, 0.99) |  | 116,475/4,881,145 | **0.98^‡^** (0.97, 0.99) | |
| White meat only | 3,628/142,888 | 0.98 (0.95, 1.01) |  | 2,308/106,927 | **0.96^*^** (0.92, 1.00) |  | 3,516/142,774 | 0.97 (0.94, 1.01) | |
| No meat/vegetarian | 1,385/56,798 | 0.99 (0.94, 1.05) |  | 939/43,988 | 1.02 (0.95, 1.08) |  | 1,342/56,755 | 0.99 (0.94, 1.04) | |
| **BMI CATEGORY** |  |  |  |  |  |  |  |  | |
| **Healthy weight** | ***n =* 509,633** |  |  | ***n =* 379,304** |  |  | ***n =* 500,684** |  | |
| Red/pr meat - High | 100,512/4,692,919 | *Reference* |  | 69,482/3,760,625 | *Reference* |  | 97,698/4,689,883 | *Reference* | |
| Red/pr meat - Moderate | 93,455/4,293,463 | **0.98^‡^** (0.97, 0.99) |  | 65,095/3,455,086 | **0.98^‡^** (0.97, 0.99) |  | 90,970/4,290,760 | **0.98^‡^** (0.97, 0.99) | |
| Red/pr meat - Low | 103,782/4,273,462 | **0.97^‡^** (0.96, 0.98) |  | 70,093/3,359,071 | **0.97^‡^** (0.96, 0.98) |  | 100,604/4,270,069 | **0.97^‡^** (0.96, 0.98) | |
| White meat only | 3,187/133,098 | **0.95^†^** (0.91, 0.98) |  | 2,026/100,529 | **0.94^†^** (0.90, 0.99) |  | 3,064/132,968 | **0.94^†^** (0.91, 0.98) | |
| No meat/vegetarian | 1,295/54,727 | 1.01 (0.95, 1.06) |  | 901/43,432 | 1.07 (1.00, 1.14) |  | 1,251/54,687 | 1.01 (0.96, 1.07) | |
| **Overweight** | ***n =* 369,608** |  |  | ***n =* 270,274** |  |  | ***n =* 363,656** |  | |
| Red/pr meat - High | 93,246/3,674,405 | *Reference* |  | 64,857/2,904,476 | *Reference* |  | 91,206/3,672,177 | *Reference* | |
| Red/pr meat - Moderate | 73,569/2,838,602 | 0.99 (0.98, 1.00) |  | 50,993/2,245,879 | **0.98^†^** (0.97, 0.99) |  | 71,904/2,836,776 | 0.99 (0.98, 1.00) | |
| Red/pr meat - Low | 74,814/2,616,677 | **0.99^*^** (0.98, 1.00) |  | 49,723/2,007,858 | **0.98^†^** (0.97, 0.99) |  | 72,787/2,614,445 | **0.99^†^** (0.98, 1.00) | |
| White meat only | 1,785/58,516 | 0.97 (0.93, 1.02) |  | 1,094/41,730 | 0.98 (0.93, 1.05) |  | 1,724/58,459 | 0.97 (0.92, 1.02) | |
| No meat/vegetarian | 702/25,259 | 0.94 (0.88, 1.02) |  | 467/19,424 | 0.97 (0.89, 1.07) |  | 674/25,231 | 0.93 (0.87, 1.01) | |
| **Obesity** | ***n =* 109,137** |  |  | ***n =* 74,733** |  |  | ***n =* 107,363** |  | |
| Red/pr meat - High | 33,025/1,163,842 | *Reference* |  | 21,569/869,501 | *Reference* |  | 32,327/1,163,063 | *Reference* | |
| Red/pr meat - Moderate | 23,116/797,396 | 1.01 (0.99, 1.02) |  | 15,169/595,781 | 1.01 (0.99, 1.03) |  | 22,631/796,871 | 1.01 (0.99, 1.02) | |
| Red/pr meat - Low | 22,806/707,088 | 1.00 (0.98, 1.01) |  | 14,278/509,500 | 1.01 (0.98, 1.03) |  | 22,260/706,489 | 1.00 (0.98, 1.02) | |
| White meat only | 523/14,713 | 0.99 (0.91, 1.08) |  | 271/9,489 | 0.90 (0.80, 1.01) |  | 503/14,693 | 0.98 (0.90, 1.07) | |
| No meat/vegetarian | 200/7,124 | 0.87 (0.76, 1.00) |  | 111/5,037 | **0.80^*^** (0.66, 0.96) |  | 194/7,116 | 0.87 (0.76, 1.01) | |
| **SMOKING STATUS** |  |  |  |  |  |  |  |  | |
| **Never smokers** | ***n =* 417,041** |  |  | ***n =* 316,670** |  |  | ***n =* 411,803** |  | |
| Red/pr meat - High | 80,783/4,128,445 | *Reference* |  | 57,661/3,338,687 | *Reference* |  | 79,257/4,126,732 | *Reference* | |
| Red/pr meat - Moderate | 72,548/3,551,808 | 0.99 (0.98, 1.00) |  | 51,785/2,881,837 | **0.98^*^** (0.97, 1.00) |  | 71,120/3,550,233 | 0.99 (0.98, 1.00) | |
| Red/pr meat - Low | 82,404/3,471,985 | 0.99 (0.98, 1.00) |  | 56,895/2,745,033 | 0.99 (0.98, 1.00) |  | 80,493/3,469,833 | 0.99 (0.98, 1.00) | |
| White meat only | 2,539/100,046 | 0.98 (0.94, 1.02) |  | 1,587/74,361 | 0.97 (0.93, 1.03) |  | 2,450/99,957 | 0.97 (0.94, 1.01) | |
| No meat/vegetarian | 1,301/56,624 | 0.97 (0.92, 1.03) |  | 903/45,245 | 1.00 (0.93, 1.06) |  | 1,259/56,581 | 0.97 (0.92, 1.02) | |
| **Former smokers** | ***n =* 293,766** |  |  | ***n =* 203,785** |  |  | ***n =* 288,080** |  | |
| Red/pr meat - High | 67,031/2,730,612 | *Reference* |  | 42,649/2,084,803 | *Reference* |  | 65,066/2,728,559 | *Reference* | |
| Red/pr meat - Moderate | 57,151/2,314,718 | **0.99^*^** (0.98, 1.00) |  | 36,584/1,778,232 | **0.98^*^** (0.97, 1.00) |  | 55,542/2,312,967 | 0.99 (0.98, 1.00) | |
| Red/pr meat - Low | 60,140/2,253,285 | **0.96^‡^** (0.95, 0.98) |  | 36,844/1,677,225 | **0.96^‡^** (0.94, 0.97) |  | 58,224/2,251,273 | **0.97^‡^** (0.95, 0.98) | |
| White meat only | 1,684/67,737 | 0.96 (0.91, 1.01) |  | 991/48,755 | 0.97 (0.91, 1.03) |  | 1,622/67,669 | 0.96 (0.91, 1.01) | |
| No meat/vegetarian | 483/18,783 | 1.08 (0.99, 1.19) |  | 304/13,753 | **1.19^†^** (1.06, 1.34) |  | 468/18,769 | **1.10^*^** (1.00, 1.20) | |
| **Ever smokers** | ***n =* 59,982** |  |  | ***n =* 42,486** |  |  | ***n =* 58,453** |  | |
| Red/pr meat - High | 14,779/538,229 | *Reference* |  | 9,969/424,073 | *Reference* |  | 14,283/537,671 | *Reference* | |
| Red/pr meat - Moderate | 11,292/410,774 | 0.98 (0.95, 1.00) |  | 7,522/320,745 | **0.97^*^** (0.94, 1.00) |  | 10,901/410,339 | **0.97^*^** (0.95, 1.00) | |
| Red/pr meat - Low | 12,675/421,313 | **0.96^†^** (0.94, 0.99) |  | 7,973/318,790 | **0.95^†^** (0.92, 0.98) |  | 12,134/420,722 | **0.96^†^** (0.94, 0.98) | |
| White meat only | 364/11,119 | 0.95 (0.85, 1.05) |  | 200/7,622 | 0.95 (0.83, 1.10) |  | 342/11,098 | 0.94 (0.84, 1.05) | |
| No meat/vegetarian | 127/3,320 | 1.00 (0.84, 1.19) |  | 68/2,309 | 1.01 (0.79, 1.28) |  | 120/3,313 | 1.01 (0.84, 1.21) | |
| **Current smokers** | ***n =* 217,589** |  |  | ***n =* 161,370** |  |  | ***n =* 213,367** |  | |
| Red/pr meat - High | 64,190/2,133,880 | *Reference* |  | 45,629/1,687,038 | *Reference* |  | 62,625/2,132,161 | *Reference* | |
| Red/pr meat - Moderate | 49,149/1,652,160 | 1.00 (0.98, 1.01) |  | 35,366/1,315,932 | 0.99 (0.98, 1.01) |  | 47,942/1,650,867 | 1.00 (0.98, 1.01) | |
| Red/pr meat - Low | 46,183/1,450,644 | 0.99 (0.97, 1.00) |  | 32,382/1,135,380 | 0.99 (0.97, 1.00) |  | 44,800/1,449,175 | **0.98^*^** (0.97, 1.00) | |
| White meat only | 908/27,424 | 0.96 (0.88, 1.03) |  | 613/21,011 | 0.93 (0.85, 1.03) |  | 877/27,396 | 0.96 (0.88, 1.04) | |
| No meat/vegetarian | 286/8,383 | 0.93 (0.81, 1.08) |  | 204/6,585 | 0.99 (0.83, 1.17) |  | 272/8,372 | 0.92 (0.80, 1.07) | |
| **AGE GROUP** |  |  |  |  |  |  |  |  | |
| **Less than 50 y** | ***n =* 257,856** |  |  | ***n =* 214,272** |  |  | ***n =* 256,891** |  | |
| Red/pr meat - High | 43,787/3,753,895 | *Reference* |  | 34,180/3,157,811 | *Reference* |  | 43,361/3,753,441 | *Reference* | |
| Red/pr meat - Moderate | 30,157/2,785,951 | 1.00 (0.98, 1.01) |  | 23,647/2,367,233 | 0.99 (0.97, 1.00) |  | 29,866/2,785,632 | 1.00 (0.98, 1.01) | |
| Red/pr meat - Low | 23,298/2,144,495 | 0.99 (0.98, 1.01) |  | 17,957/1,804,592 | 0.99 (0.97, 1.01) |  | 23,059/2,144,240 | 0.99 (0.98, 1.01) | |
| White meat only | 525/56,641 | 0.97 (0.89, 1.06) |  | 366/46,935 | **0.89^*^** (0.80, 0.99) |  | 517/56,632 | 0.97 (0.89, 1.06) | |
| No meat/vegetarian | 266/28,825 | 1.09 (0.96, 1.23) |  | 208/24,804 | 1.06 (0.93, 1.22) |  | 265/28,823 | 1.09 (0.97, 1.23) | |
| **50 to 64 y** | ***n =* 514,981** |  |  | ***n =* 381,342** |  |  | ***n =* 509,019** |  | |
| Red/pr meat - High | 135,375/4,887,174 | *Reference* |  | 95,863/3,798,042 | *Reference* |  | 133,119/4,884,793 | *Reference* | |
| Red/pr meat - Moderate | 113,914/4,240,937 | **0.99^†^** (0.98, 1.00) |  | 81,613/3,329,630 | **0.98^‡^** (0.97, 0.99) |  | 112,181/4,239,095 | **0.99^†^** (0.98, 1.00) | |
| Red/pr meat - Low | 113,628/4,188,521 | **0.97^‡^** (0.97, 0.98) |  | 79,662/3,231,288 | **0.97^‡^** (0.96, 0.98) |  | 111,724/4,186,489 | **0.97^‡^** (0.97, 0.98) | |
| White meat only | 2,791/108,441 | **0.94^‡^** (0.90, 0.97) |  | 1,796/78,112 | **0.92^‡^** (0.88, 0.97) |  | 2,735/108,381 | **0.93^‡^** (0.90, 0.97) | |
| No meat/vegetarian | 993/40,492 | **0.94^*^** (0.88, 1.00) |  | 724/31,276 | 0.98 (0.91, 1.05) |  | 980/40,481 | **0.94^*^** (0.88, 1.00) | |
| **65 y or more** | ***n =* 215,541** |  |  | ***n =* 128,697** |  |  | ***n =* 205,793** |  | |
| Red/pr meat - High | 47,621/890,097 | *Reference* |  | 25,865/578,749 | *Reference* |  | 44,751/886,889 | *Reference* | |
| Red/pr meat - Moderate | 46,069/902,573 | **0.98^†^** (0.97, 0.99) |  | 25,997/599,884 | 0.98 (0.97, 1.00) |  | 43,458/899,680 | **0.98^†^** (0.97, 0.99) | |
| Red/pr meat - Low | 64,476/1,264,210 | **0.97^‡^** (0.96, 0.98) |  | 36,475/840,549 | **0.97^‡^** (0.96, 0.99) |  | 60,868/1,260,274 | **0.97^‡^** (0.96, 0.98) | |
| White meat only | 2,179/41,245 | 0.99 (0.95, 1.03) |  | 1,229/26,701 | 1.02 (0.96, 1.08) |  | 2,039/41,108 | 0.98 (0.94, 1.03) | |
| No meat/vegetarian | 938/17,793 | 0.98 (0.92, 1.05) |  | 547/11,813 | 1.03 (0.95, 1.13) |  | 874/17,730 | 0.98 (0.91, 1.04) | |
| **ATTAINED AGE** |  |  |  |  |  |  |  |  | |
| **Less than 80 y** | ***n =* 972,717** |  |  | ***n =* 716,486** |  |  | ***n =* 958,198** |  | |
| Red/pr meat - High | 106,100/7,818,882 | *Reference* |  | 67,898/6,197,708 | *Reference* |  | 101,179/7,813,597 | *Reference* | |
| Red/pr meat - Moderate | 81,658/6,352,383 | **0.99^*^** (0.98, 1.00) |  | 51,947/5,061,326 | **0.98^‡^** (0.97, 0.99) |  | 77,551/6,347,974 | **0.99^†^** (0.98, 1.00) | |
| Red/pr meat - Low | 80,077/5,853,832 | **0.98^‡^** (0.97, 0.99) |  | 48,258/4,546,120 | **0.97^‡^** (0.96, 0.99) |  | 75,150/5,848,588 | **0.98^‡^** (0.97, 0.99) | |
| White meat only | 2,102/155,546 | 1.00 (0.96, 1.05) |  | 1,138/115,041 | 0.98 (0.93, 1.04) |  | 1,935/155,377 | 0.99 (0.95, 1.04) | |
| No meat/vegetarian | 809/66,214 | 1.02 (0.95, 1.10) |  | 503/52,401 | 1.05 (0.97, 1.15) |  | 761/66,170 | 1.04 (0.97, 1.12) | |
| **80 y or more** | ***n =* 668,289** |  |  | ***n =* 511,861** |  |  | ***n =* 665,784** |  | |
| Red/pr meat - High | 120,683/1,712,284 | *Reference* |  | 88,010/1,336,894 | *Reference* |  | 120,052/1,711,525 | *Reference* | |
| Red/pr meat - Moderate | 108,482/1,577,077 | **0.99^*^** (0.98, 1.00) |  | 79,310/1,235,421 | **0.99^*^** (0.98, 1.00) |  | 107,954/1,576,432 | **0.99^*^** (0.98, 1.00) | |
| Red/pr meat - Low | 121,325/1,743,394 | **0.98^‡^** (0.97, 0.98) |  | 85,836/1,330,309 | **0.98^‡^** (0.97, 0.98) |  | 120,501/1,742,414 | **0.98^‡^** (0.97, 0.98) | |
| White meat only | 3,393/50,781 | **0.94^‡^** (0.91, 0.97) |  | 2,253/36,707 | **0.94^†^** (0.90, 0.98) |  | 3,356/50,743 | **0.94^‡^** (0.91, 0.97) | |
| No meat/vegetarian | 1,388/20,896 | 0.95 (0.90, 1.00) |  | 976/15,492 | 0.99 (0.93, 1.06) |  | 1,358/20,865 | **0.95^*^** (0.90, 1.00) | |

HR (95% CI) from Cox proportional hazards models with statistically significant results in bold: ^*^ *P* < 0.05, ^†^ *P* < 0.01, ^‡^ *P* < 0.001; pr, processed

^1^ Subgroups are based on formal interaction tests showing significant effect modification (otherwise, cells are left blank); The interactions tested were each added separately to Model 5 as follows: diet^*^sex, diet^*^simple BMI category, diet^*^simple smoking status, diet^*^age group, diet^*^attained age.

^2^ Model 5 (fully adjusted) = mortality outcome ~ exposure + strata (age) + demographic factors demographics (sex [not adjusted in sex strata], race, education level, marital status, U.S. Census Bureau region) + lifestyle factors (12-level smoking status [not adjusted in smoking strata; replaced with years since quit (*Former* *smokers*) or cigarettes per day with years (*Current smokers*)], alcohol intake, physical activity, sleep) + health history (number of comorbidities [not adjusted in healthy cohort], high blood pressure) + 6-level BMI (continuous BMI for BMI strata)

^3^ Exclusions were as follows: History of heart diseases, cancers (except for non-melanoma skin cancers), stroke, chronic respiratory conditions (emphysema, chronic bronchitis, or asthma), diabetes mellitus, chronic kidney diseases, chronic liver diseases.

Supplemental Table 5. Risk of heart disease mortality by diet categories and stratification by subgroups^1^ with results from fully adjusted models^2^ in the main analysis and sensitivity analyses

|  | **Full analytic cohort**  *n =* 988,378 | |  | **Healthy cohort^3^**  *n =* 724,311 | |  | **Excludes first 2 y of follow-up**  *n =* 971,703 | |
| --- | --- | --- | --- | --- | --- | --- | --- | --- |
| **Participants analyzed** | **Deaths/person-years** | **Fully adjusted model** |  | **Deaths/person-years** | **Fully adjusted model** |  | **Deaths/person-years** | **Fully adjusted model** |
| **BMI CATEGORY** |  |  |  | No significant interactions^1^ | |  |  |  |
| **Healthy weight** | ***n =* 509,633** |  |  |  |  |  | ***n =* 500,684** |  |
| Red/pr meat - High | 24,474/4,692,919 | *Reference* |  |  |  |  | 23,553/4,689,883 | *Reference* |
| Red/pr meat - Moderate | 23,183/4,293,463 | **0.97^‡^** (0.95, 0.99) |  |  |  |  | 22,349/4,290,760 | **0.97^‡^** (0.95, 0.99) |
| Red/pr meat - Low | 27,907/4,273,462 | **0.97^‡^** (0.95, 0.99) |  |  |  |  | 26,772/4,270,069 | **0.97^‡^** (0.95, 0.98) |
| White meat only | 933/133,098 | 0.96 (0.90, 1.03) |  |  |  |  | 878/132,968 | 0.95 (0.89, 1.02) |
| No meat/vegetarian | 375/54,727 | 1.03 (0.93, 1.14) |  |  |  |  | 354/54,687 | 1.03 (0.92, 1.14) |
| **Overweight** | ***n =* 369,608** |  |  |  |  |  | ***n =* 363,656** |  |
| Red/pr meat - High | 27,383/3,674,405 | *Reference* |  |  |  |  | 26,550/3,672,177 | *Reference* |
| Red/pr meat - Moderate | 22,328/2,838,602 | 1.00 (0.98, 1.02) |  |  |  |  | 21,640/2,836,776 | 1.00 (0.98, 1.02) |
| Red/pr meat - Low | 24,169/2,616,677 | 1.01 (0.99, 1.03) |  |  |  |  | 23,332/2,614,445 | 1.01 (0.99, 1.03) |
| White meat only | 615/58,516 | 1.00 (0.92, 1.08) |  |  |  |  | 589/58,459 | 1.00 (0.92, 1.08) |
| No meat/vegetarian | 230/25,259 | 0.96 (0.84, 1.09) |  |  |  |  | 217/25,231 | 0.94 (0.82, 1.08) |
| **Obesity** | ***n =* 109,137** |  |  |  |  |  | ***n =* 107,363** |  |
| Red/pr meat - High | 11,011/1,163,842 | *Reference* |  |  |  |  | 10,718/1,163,063 | *Reference* |
| Red/pr meat - Moderate | 7,625/797,396 | 0.99 (0.96, 1.01) |  |  |  |  | 7,402/796,871 | 0.98 (0.95, 1.01) |
| Red/pr meat - Low | 7,957/707,088 | 1.00 (0.97, 1.03) |  |  |  |  | 7,721/706,489 | 1.00 (0.97, 1.03) |
| White meat only | 206/14,713 | 1.08 (0.94, 1.24) |  |  |  |  | 198/14,693 | 1.08 (0.94, 1.24) |
| No meat/vegetarian | 73/7,124 | 0.90 (0.72, 1.14) |  |  |  |  | 69/7,116 | 0.88 (0.70, 1.12) |
| **SMOKING STATUS** |  |  |  | No significant interactions^1^ | |  |  |  |
| **Never smokers** | ***n =* 417,041** |  |  |  |  |  | ***n =* 411,803** |  |
| Red/pr meat - High | 22,759/4,128,445 | *Reference* |  |  |  |  | 22,218/4,126,732 | *Reference* |
| Red/pr meat - Moderate | 20,796/3,551,808 | 0.99 (0.97, 1.01) |  |  |  |  | 20,258/3,550,233 | 0.99 (0.97, 1.01) |
| Red/pr meat - Low | 25,384/3,471,985 | 1.00 (0.98, 1.02) |  |  |  |  | 24,654/3,469,833 | 1.00 (0.98, 1.02) |
| White meat only | 828/100,046 | 0.99 (0.93, 1.07) |  |  |  |  | 784/99,957 | 0.98 (0.91, 1.05) |
| No meat/vegetarian | 407/56,624 | 0.98 (0.89, 1.09) |  |  |  |  | 391/56,581 | 0.98 (0.89, 1.09) |
| **Former smokers** | ***n =* 293,766** |  |  |  |  |  | ***n =* 288,080** |  |
| Red/pr meat - High | 19,315/2,730,612 | *Reference* |  |  |  |  | 18,591/2,728,559 | *Reference* |
| Red/pr meat - Moderate | 16,753/2,314,718 | 0.99 (0.97, 1.01) |  |  |  |  | 16,143/2,312,967 | 0.99 (0.97, 1.02) |
| Red/pr meat - Low | 18,345/2,253,285 | **0.97^†^** (0.95, 0.99) |  |  |  |  | 17,572/2,251,273 | **0.97^†^** (0.95, 0.99) |
| White meat only | 561/67,737 | 1.03 (0.94, 1.12) |  |  |  |  | 535/67,669 | 1.03 (0.94, 1.12) |
| No meat/vegetarian | 142/18,783 | 1.10 (0.93, 1.30) |  |  |  |  | 134/18,769 | 1.09 (0.92, 1.30) |
| **Ever smokers** | ***n =* 59,982** |  |  |  |  |  | ***n =* 58,453** |  |
| Red/pr meat - High | 4,649/538,229 | *Reference* |  |  |  |  | 4,447/537,671 | *Reference* |
| Red/pr meat - Moderate | 3,503/410,774 | **0.95^*^** (0.91, 0.99) |  |  |  |  | 3,347/410,339 | **0.95^*^** (0.91, 0.99) |
| Red/pr meat - Low | 4,311/421,313 | 0.98 (0.94, 1.02) |  |  |  |  | 4,093/420,722 | 0.98 (0.94, 1.02) |
| White meat only | 119/11,119 | 0.90 (0.75, 1.08) |  |  |  |  | 110/11,098 | 0.88 (0.73, 1.07) |
| No meat/vegetarian | 49/3,320 | 1.06 (0.80, 1.41) |  |  |  |  | 44/3,313 | 1.03 (0.76, 1.39) |
| **Current smokers** | ***n =* 217,589** |  |  |  |  |  | ***n =* 213,367** |  |
| Red/pr meat - High | 16,145/2,133,880 | *Reference* |  |  |  |  | 15,565/2,132,161 | *Reference* |
| Red/pr meat - Moderate | 12,084/1,652,160 | 0.99 (0.96, 1.01) |  |  |  |  | 11,643/1,650,867 | 0.98 (0.96, 1.01) |
| Red/pr meat - Low | 11,993/1,450,644 | 0.99 (0.96, 1.01) |  |  |  |  | 11,506/1,449,175 | 0.98 (0.96, 1.01) |
| White meat only | 246/27,424 | 0.96 (0.82, 1.12) |  |  |  |  | 236/27,396 | 0.97 (0.83, 1.14) |
| No meat/vegetarian | 80/8,383 | 0.87 (0.65, 1.15) |  |  |  |  | 71/8,372 | 0.79 (0.59, 1.07) |
| **AGE GROUP** |  |  |  | No significant interactions^1^ | |  |  |  |
| **Less than 50 y** | ***n =* 257,856** |  |  |  |  |  | ***n =* 256,891** |  |
| Red/pr meat - High | 9,437/3,753,895 | *Reference* |  |  |  |  | 9,329/3,753,441 | *Reference* |
| Red/pr meat - Moderate | 6,247/2,785,951 | 1.00 (0.97, 1.03) |  |  |  |  | 6,164/2,785,632 | 1.00 (0.97, 1.03) |
| Red/pr meat - Low | 5,161/2,144,495 | **1.05^†^** (1.02, 1.09) |  |  |  |  | 5,102/2,144,240 | **1.05^†^** (1.02, 1.09) |
| White meat only | 115/56,641 | 1.05 (0.87, 1.26) |  |  |  |  | 114/56,632 | 1.05 (0.88, 1.27) |
| No meat/vegetarian | 46/28,825 | 0.94 (0.70, 1.26) |  |  |  |  | 45/28,823 | 0.93 (0.69, 1.25) |
| **50 to 64 y** | ***n =* 514,981** |  |  |  |  |  | ***n =* 509,019** |  |
| Red/pr meat - High | 36,556/4,887,174 | *Reference* |  |  |  |  | 35,763/4,884,793 | *Reference* |
| Red/pr meat - Moderate | 30,337/4,240,937 | **0.98^*^** (0.97, 1.00) |  |  |  |  | 29,720/4,239,095 | **0.98^*^** (0.97, 1.00) |
| Red/pr meat - Low | 31,403/4,188,521 | **0.98^*^** (0.97, 1.00) |  |  |  |  | 30,735/4,186,489 | **0.98^*^** (0.97, 1.00) |
| White meat only | 779/108,441 | 0.94 (0.87, 1.01) |  |  |  |  | 758/108,381 | 0.93 (0.87, 1.00) |
| No meat/vegetarian | 271/40,492 | 0.94 (0.84, 1.06) |  |  |  |  | 268/40,481 | 0.95 (0.84, 1.07) |
| **65 y or more** | ***n =* 215,541** |  |  |  |  |  | ***n =* 205,793** |  |
| Red/pr meat - High | 16,875/890,097 | *Reference* |  |  |  |  | 15,729/886,889 | *Reference* |
| Red/pr meat - Moderate | 16,552/902,573 | 0.98 (0.96, 1.00) |  |  |  |  | 15,507/899,680 | 0.98 (0.96, 1.00) |
| Red/pr meat - Low | 23,469/1,264,210 | **0.97^†^** (0.95, 0.99) |  |  |  |  | 21,988/1,260,274 | **0.97^‡^** (0.95, 0.99) |
| White meat only | 860/41,245 | 1.03 (0.96, 1.10) |  |  |  |  | 793/41,108 | 1.01 (0.94, 1.09) |
| No meat/vegetarian | 361/17,793 | 1.03 (0.92, 1.14) |  |  |  |  | 327/17,730 | 1.00 (0.90, 1.12) |
| **ATTAINED AGE** |  |  |  |  |  |  |  |  |
| **Less than 80 y** | ***n =* 972,717** |  |  | ***n =* 716,486** |  |  | ***n =* 958,198** |  |
| Red/pr meat - High | 28,439/7,818,882 | *Reference* |  | 16,153/6,197,708 | *Reference* |  | 26,664/7,813,597 | *Reference* |
| Red/pr meat - Moderate | 22,004/6,352,383 | 0.99 (0.98, 1.01) |  | 12,113/5,061,326 | 0.98 (0.96, 1.00) |  | 20,488/6,347,974 | 0.99 (0.97, 1.01) |
| Red/pr meat - Low | 22,952/5,853,832 | 0.99 (0.97, 1.01) |  | 11,809/4,546,120 | 0.99 (0.97, 1.02) |  | 21,099/5,848,588 | 0.99 (0.97, 1.01) |
| White meat only | 628/155,546 | 1.01 (0.93, 1.09) |  | 276/115,041 | 0.98 (0.87, 1.11) |  | 559/155,377 | 0.99 (0.91, 1.08) |
| No meat/vegetarian | 224/66,214 | 1.01 (0.89, 1.15) |  | 115/52,401 | 1.01 (0.84, 1.21) |  | 204/66,170 | 1.02 (0.89, 1.17) |
| **80 y or more** | ***n =* 668,289** |  |  | ***n =* 511,861** |  |  | ***n =* 665,784** |  |
| Red/pr meat - High | 34,429/1,712,284 | *Reference* |  | 23,796/1,336,894 | *Reference* |  | 34,157/1,711,525 | *Reference* |
| Red/pr meat - Moderate | 31,132/1,577,077 | **0.98^†^** (0.96, 0.99) |  | 21,532/1,235,421 | **0.98^*^** (0.96, 1.00) |  | 30,903/1,576,432 | **0.98^†^** (0.96, 0.99) |
| Red/pr meat - Low | 37,081/1,743,394 | **0.98^†^** (0.97, 1.00) |  | 24,687/1,330,309 | 0.98 (0.97, 1.00) |  | 36,726/1,742,414 | **0.98^*^** (0.97, 1.00) |
| White meat only | 1,126/50,781 | 0.98 (0.92, 1.04) |  | 702/36,707 | 1.00 (0.93, 1.08) |  | 1,106/50,743 | 0.98 (0.92, 1.04) |
| No meat/vegetarian | 454/20,896 | 0.98 (0.90, 1.08) |  | 306/15,492 | 1.06 (0.95, 1.19) |  | 436/20,865 | 0.97 (0.88, 1.06) |

HR (95% CI) from Cox proportional hazards models with statistically significant results in bold: ^*^ *P* < 0.05, ^†^ *P* < 0.01, ^‡^ *P* < 0.001; pr, processed

^1^ Subgroups are based on formal interaction tests showing significant effect modification (otherwise, cells are left blank); The interactions tested were each added separately to Model 5 as follows: diet^*^sex, diet^*^simple BMI category, diet^*^simple smoking status, diet^*^age group, diet^*^attained age.

^2^ Model 5 (fully adjusted) = mortality outcome ~ exposure + strata (age) + demographic factors demographics (sex [not adjusted in sex strata], race, education level, marital status, U.S. Census Bureau region) + lifestyle factors (12-level smoking status [not adjusted in smoking strata; replaced with years since quit (*Former* *smokers*) or cigarettes per day with years (*Current smokers*)], alcohol intake, physical activity, sleep) + health history (history of heart disease [not adjusted in healthy cohort], high blood pressure, menopause status, hormone replacement therapy) + 6-level BMI (continuous BMI for BMI strata)

^3^ Exclusions were as follows: History of heart diseases, cancers (except for non-melanoma skin cancers), stroke, chronic respiratory conditions (emphysema, chronic bronchitis, or asthma), diabetes mellitus, chronic kidney diseases, chronic liver diseases.

Supplemental Table 6. Risk of cancer mortality by diet categories and stratification by subgroups^1^ with results from fully adjusted models^2^ in the main analysis and sensitivity analyses

|  | **Full analytic cohort**  *n =* 988,378 | |  | **Healthy cohort^3^**  *n =* 724,311 | |  | **Excludes first 2 y of follow-up**  *n =* 971,703 | |
| --- | --- | --- | --- | --- | --- | --- | --- | --- |
| **Participants analyzed** | **Deaths/person-years** | **Fully adjusted model** |  | **Deaths/person-years** | **Fully adjusted model** |  | **Deaths/person-years** | **Fully adjusted model** |
| **SEX** |  |  |  |  |  |  |  |  |
| **Males** | ***n =* 442,437** |  |  | ***n =* 318,434** |  |  | ***n =* 431,892** |  |
| Red/pr meat - High | 35,131/4,418,143 | *Reference* |  | 25,311/3,504,121 | *Reference* |  | 33,817/4,414,021 | *Reference* |
| Red/pr meat - Moderate | 24,360/3,076,934 | 0.98 (0.97, 1.00) |  | 17,474/2,443,164 | 0.98 (0.96, 1.00) |  | 23,360/3,073,817 | **0.98^*^** (0.97, 1.00) |
| Red/pr meat - Low | 22,243/2,713,499 | **0.98^*^** (0.96, 1.00) |  | 15,407/2,080,390 | 0.98 (0.96, 1.00) |  | 21,203/2,709,858 | **0.98^*^** (0.96, 1.00) |
| White meat only | 476/63,439 | 0.96 (0.87, 1.05) |  | 288/44,821 | 0.93 (0.82, 1.04) |  | 455/63,346 | 0.97 (0.88, 1.06) |
| No meat/vegetarian | 209/30,311 | 0.95 (0.83, 1.09) |  | 141/23,905 | 0.97 (0.82, 1.14) |  | 201/30,280 | 0.98 (0.85, 1.12) |
| **Females** | ***n =* 545,941** |  |  | ***n =* 405,877** |  |  | ***n =* 539,811** |  |
| Red/pr meat - High | 25,668/5,113,024 | *Reference* |  | 18,023/4,030,481 | *Reference* |  | 24,887/5,111,102 | *Reference* |
| Red/pr meat - Moderate | 25,451/4,852,527 | 1.00 (0.98, 1.02) |  | 17,865/3,853,583 | 0.99 (0.97, 1.01) |  | 24,671/4,850,590 | 1.00 (0.98, 1.02) |
| Red/pr meat - Low | 27,347/4,883,727 | **0.98^*^** (0.97, 1.00) |  | 18,625/3,796,039 | **0.98^*^** (0.96, 1.00) |  | 26,348/4,881,145 | 0.98 (0.97, 1.00) |
| White meat only | 804/142,888 | 0.99 (0.92, 1.06) |  | 504/106,927 | 0.95 (0.87, 1.04) |  | 757/142,774 | 0.97 (0.90, 1.05) |
| No meat/vegetarian | 303/56,798 | 1.02 (0.91, 1.14) |  | 210/43,988 | 1.05 (0.92, 1.21) |  | 289/56,755 | 1.02 (0.91, 1.15) |
| **SMOKING STATUS** |  |  |  | No significant interactions^1^ | |  |  |  |
| **Never smokers** | ***n =* 417,041** |  |  |  |  |  | ***n =* 411,803** |  |
| Red/pr meat - High | 18,175/4,128,445 | *Reference* |  |  |  |  | 17,613/4,126,732 | *Reference* |
| Red/pr meat - Moderate | 16,108/3,551,808 | 1.00 (0.98, 1.02) |  |  |  |  | 15,566/3,550,233 | 1.00 (0.98, 1.02) |
| Red/pr meat - Low | 17,066/3,471,985 | 0.99 (0.97, 1.02) |  |  |  |  | 16,401/3,469,833 | 1.00 (0.97, 1.02) |
| White meat only | 540/100,046 | 1.05 (0.97, 1.15) |  |  |  |  | 512/99,957 | 1.05 (0.96, 1.15) |
| No meat/vegetarian | 271/56,624 | 0.98 (0.87, 1.11) |  |  |  |  | 258/56,581 | 0.98 (0.87, 1.11) |
| **Former smokers** | ***n =* 293,766** |  |  |  |  |  | ***n =* 288,080** |  |
| Red/pr meat - High | 17,952/2,730,612 | *Reference* |  |  |  |  | 17,176/2,728,559 | *Reference* |
| Red/pr meat - Moderate | 15,031/2,314,718 | 0.99 (0.97, 1.01) |  |  |  |  | 14,398/2,312,967 | 0.99 (0.97, 1.01) |
| Red/pr meat - Low | 15,268/2,253,285 | **0.97^†^** (0.95, 0.99) |  |  |  |  | 14,580/2,251,273 | **0.97^*^** (0.95, 1.00) |
| White meat only | 405/67,737 | 0.94 (0.85, 1.04) |  |  |  |  | 383/67,669 | 0.94 (0.84, 1.04) |
| No meat/vegetarian | 127/18,783 | 1.13 (0.95, 1.35) |  |  |  |  | 123/18,769 | 1.17 (0.98, 1.40) |
| **Ever smokers** | ***n =* 59,982** |  |  |  |  |  | ***n =* 58,453** |  |
| Red/pr meat - High | 3,826/538,229 | *Reference* |  |  |  |  | 3,662/537,671 | *Reference* |
| Red/pr meat - Moderate | 2,973/410,774 | 1.00 (0.95, 1.05) |  |  |  |  | 2,832/410,339 | 1.00 (0.95, 1.05) |
| Red/pr meat - Low | 3,098/421,313 | **0.95^*^** (0.91, 1.00) |  |  |  |  | 2,918/420,722 | **0.94^*^** (0.90, 0.99) |
| White meat only | 82/11,119 | 0.91 (0.73, 1.13) |  |  |  |  | 77/11,098 | 0.92 (0.73, 1.15) |
| No meat/vegetarian | 29/3,320 | 1.00 (0.69, 1.44) |  |  |  |  | 28/3,313 | 1.05 (0.72, 1.52) |
| **Current smokers** | ***n =* 217,589** |  |  |  |  |  | ***n =* 213,367** |  |
| Red/pr meat - High | 20,846/2,133,880 | *Reference* |  |  |  |  | 20,253/2,132,161 | *Reference* |
| Red/pr meat - Moderate | 15,699/1,652,160 | 1.00 (0.98, 1.02) |  |  |  |  | 15,235/1,650,867 | 1.00 (0.98, 1.02) |
| Red/pr meat - Low | 14,158/1,450,644 | 1.00 (0.97, 1.02) |  |  |  |  | 13,652/1,449,175 | 0.99 (0.97, 1.02) |
| White meat only | 253/27,424 | 0.90 (0.77, 1.05) |  |  |  |  | 240/27,396 | 0.90 (0.78, 1.05) |
| No meat/vegetarian | 85/8,383 | 1.03 (0.80, 1.33) |  |  |  |  | 81/8,372 | 1.04 (0.81, 1.35) |
| **AGE GROUP** |  |  |  |  |  |  |  |  |
| **Less than 50 y** | ***n =* 257,856** |  |  | ***n =* 214,272** |  |  | ***n =* 256,891** |  |
| Red/pr meat - High | 14,457/3,753,895 | *Reference* |  | 11,591/3,157,811 | *Reference* |  | 14,246/3,753,441 | *Reference* |
| Red/pr meat - Moderate | 10,243/2,785,951 | 1.00 (0.98, 1.03) |  | 8,159/2,367,233 | 0.99 (0.96, 1.02) |  | 10,108/2,785,632 | 1.00 (0.98, 1.03) |
| Red/pr meat - Low | 7,548/2,144,495 | 0.97 (0.95, 1.00) |  | 6,000/1,804,592 | 0.97 (0.94, 1.01) |  | 7,445/2,144,240 | 0.98 (0.95, 1.00) |
| White meat only | 195/56,641 | 1.10 (0.96, 1.27) |  | 138/46,935 | 1.01 (0.85, 1.19) |  | 189/56,632 | 1.09 (0.94, 1.26) |
| No meat/vegetarian | 98/28,825 | 1.22 (1.00, 1.49) |  | 77/24,804 | 1.18 (0.94, 1.48) |  | 98/28,823 | **1.24^*^** (1.02, 1.52) |
| **50 to 64 y** | ***n =* 514,981** |  |  | ***n =* 381,342** |  |  | ***n =* 509,019** |  |
| Red/pr meat - High | 35,878/4,887,174 | *Reference* |  | 25,972/3,798,042 | *Reference* |  | 34,905/4,884,793 | *Reference* |
| Red/pr meat - Moderate | 29,622/4,240,937 | 0.99 (0.97, 1.00) |  | 21,547/3,329,630 | 0.99 (0.97, 1.00) |  | 28,861/4,239,095 | 0.99 (0.97, 1.00) |
| Red/pr meat - Low | 28,718/4,188,521 | **0.98^†^** (0.96, 0.99) |  | 20,436/3,231,288 | **0.98^*^** (0.96, 1.00) |  | 27,915/4,186,489 | **0.98^*^** (0.96, 1.00) |
| White meat only | 689/108,441 | 0.96 (0.89, 1.03) |  | 440/78,112 | 0.93 (0.85, 1.02) |  | 663/108,381 | 0.95 (0.88, 1.03) |
| No meat/vegetarian | 235/40,492 | 0.93 (0.82, 1.06) |  | 158/31,276 | 0.90 (0.77, 1.06) |  | 227/40,481 | 0.93 (0.82, 1.06) |
| **65 y or more** | ***n =* 215,541** |  |  | ***n =* 128,697** |  |  | ***n =* 205,793** |  |
| Red/pr meat - High | 10,464/890,097 | *Reference* |  | 5,771/578,749 | *Reference* |  | 9,553/886,889 | *Reference* |
| Red/pr meat - Moderate | 9,946/902,573 | 0.99 (0.96, 1.02) |  | 5,633/599,884 | 0.99 (0.95, 1.03) |  | 9,062/899,680 | 0.98 (0.95, 1.01) |
| Red/pr meat - Low | 13,324/1,264,210 | 0.98 (0.95, 1.00) |  | 7,596/840,549 | 0.98 (0.95, 1.02) |  | 12,191/1,260,274 | 0.98 (0.95, 1.00) |
| White meat only | 396/41,245 | 0.94 (0.85, 1.04) |  | 214/26,701 | 0.92 (0.80, 1.06) |  | 360/41,108 | 0.94 (0.84, 1.04) |
| No meat/vegetarian | 179/17,793 | 0.96 (0.83, 1.12) |  | 116/11,813 | 1.12 (0.93, 1.35) |  | 165/17,730 | 0.99 (0.85, 1.15) |
| **ATTAINED AGE** |  |  |  | No significant interactions^1^ | |  |  |  |
| **Less than 80 y** | ***n =* 972,717** |  |  |  |  |  | ***n =* 958,198** |  |
| Red/pr meat - High | 38,920/7,818,882 | *Reference* |  |  |  |  | 36,956/7,813,597 | *Reference* |
| Red/pr meat - Moderate | 30,410/6,352,383 | **0.98^*^** (0.97, 1.00) |  |  |  |  | 28,754/6,347,974 | **0.98^*^** (0.97, 1.00) |
| Red/pr meat - Low | 28,809/5,853,832 | **0.97^‡^** (0.96, 0.99) |  |  |  |  | 26,954/5,848,588 | **0.97^†^** (0.96, 0.99) |
| White meat only | 752/155,546 | 1.02 (0.95, 1.10) |  |  |  |  | 692/155,377 | 1.02 (0.95, 1.10) |
| No meat/vegetarian | 292/66,214 | 1.03 (0.92, 1.16) |  |  |  |  | 274/66,170 | 1.06 (0.94, 1.19) |
| **80 y or more** | ***n =* 668,289** |  |  |  |  |  | ***n =* 665,784** |  |
| Red/pr meat - High | 21,879/1,712,284 | *Reference* |  |  |  |  | 21,748/1,711,525 | *Reference* |
| Red/pr meat - Moderate | 19,401/1,577,077 | 1.00 (0.99, 1.02) |  |  |  |  | 19,277/1,576,432 | 1.00 (0.98, 1.02) |
| Red/pr meat - Low | 20,781/1,743,394 | 0.99 (0.97, 1.01) |  |  |  |  | 20,597/1,742,414 | 0.99 (0.97, 1.01) |
| White meat only | 528/50,781 | **0.91^*^** (0.83, 0.99) |  |  |  |  | 520/50,743 | **0.91^*^** (0.83, 0.99) |
| No meat/vegetarian | 220/20,896 | 0.94 (0.82, 1.07) |  |  |  |  | 216/20,865 | 0.94 (0.82, 1.08) |

HR (95% CI) from Cox proportional hazards models with statistically significant results in bold: ^*^ *P* < 0.05, ^†^ *P* < 0.01, ^‡^ *P* < 0.001; pr, processed

^1^ Subgroups are based on formal interaction tests showing significant effect modification (otherwise, cells are left blank); The interactions tested were each added separately to Model 5 as follows: diet^*^sex, diet^*^simple BMI category, diet^*^simple smoking status, diet^*^age group, diet^*^attained age.

^2^ Model 5 (fully adjusted) = mortality outcome ~ exposure + strata (age) + demographic factors demographics (sex [not adjusted in sex strata], race, education level, marital status, U.S. Census Bureau region) + lifestyle factors (12-level smoking status [not adjusted in smoking strata; replaced with years since quit (*Former* *smokers*) or cigarettes per day with years (*Current smokers*)], alcohol intake, physical activity, sleep) + health history (history of cancer [not adjusted in healthy cohort], family history of cancer, history of diabetes [not adjusted in healthy cohort], menopause status, hormone replacement therapy) + 6-level BMI (continuous BMI for BMI strata)

^3^ Exclusions were as follows: History of heart diseases, cancers (except for non-melanoma skin cancers), stroke, chronic respiratory conditions (emphysema, chronic bronchitis, or asthma), diabetes mellitus, chronic kidney diseases, chronic liver diseases.

Supplemental Table 7. Risk of stroke mortality by diet categories and stratification by subgroups^1^ with results from fully adjusted models^2^ in the main analysis and sensitivity analyses

|  | **Full analytic cohort**  *n =* 988,378 | |  | **Healthy cohort^3^**  *n =* 724,311 | |  | **Excludes first 2 y of follow-up**  *n =* 971,703 | |
| --- | --- | --- | --- | --- | --- | --- | --- | --- |
| **Participants analyzed** | **Deaths/person-years** | **Fully adjusted model** |  | **Deaths/person-years** | **Fully adjusted model** |  | **Deaths/person-years** | **Fully adjusted model** |
| **SEX** | No significant interactions^1^ | |  |  |  |  | No significant interactions^1^ | |
| **Males** |  |  |  | ***n =* 318,434** |  |  |  |  |
| Red/pr meat - High |  |  |  | 4,723/3,504,121 | *Reference* |  |  |  |
| Red/pr meat - Moderate |  |  |  | 3,577/2,443,164 | 1.02 (0.98, 1.07) |  |  |  |
| Red/pr meat - Low |  |  |  | 3,252/2,080,390 | 0.98 (0.94, 1.03) |  |  |  |
| White meat only |  |  |  | 61/44,821 | 0.84 (0.65, 1.08) |  |  |  |
| No meat/vegetarian |  |  |  | 53/23,905 | **1.50^†^** (1.14, 1.97) |  |  |  |
| **Females** |  |  |  | ***n =* 405,877** |  |  |  |  |
| Red/pr meat - High |  |  |  | 5,728/4,030,481 | *Reference* |  |  |  |
| Red/pr meat - Moderate |  |  |  | 5,927/3,853,583 | 0.99 (0.96, 1.03) |  |  |  |
| Red/pr meat - Low |  |  |  | 6,851/3,796,039 | 0.97 (0.93, 1.00) |  |  |  |
| White meat only |  |  |  | 200/106,927 | 0.93 (0.80, 1.07) |  |  |  |
| No meat/vegetarian |  |  |  | 91/43,988 | 1.06 (0.86, 1.31) |  |  |  |
| **SMOKING STATUS**^4^ | No significant interactions^1^ | |  | No significant interactions^1^ | |  |  |  |
| **Never smokers** |  |  |  |  |  |  | ***n =* 411,803** |  |
| Red/pr meat - High |  |  |  |  |  |  | 6,456/4,126,732 | *Reference* |
| Red/pr meat - Moderate |  |  |  |  |  |  | 6,086/3,550,233 | 1.00 (0.97, 1.04) |
| Red/pr meat - Low |  |  |  |  |  |  | 6,983/3,469,833 | 0.98 (0.95, 1.02) |
| White meat only |  |  |  |  |  |  | 205/99,957 | 0.90 (0.78, 1.03) |
| No meat/vegetarian |  |  |  |  |  |  | 129/56,581 | 1.10 (0.92, 1.31) |
| **Former smokers** |  |  |  |  |  |  | ***n =* 288,080** |  |
| Red/pr meat - High |  |  |  |  |  |  | 4,100/2,728,559 | *Reference* |
| Red/pr meat - Moderate |  |  |  |  |  |  | 3,670/2,312,967 | 0.99 (0.95, 1.04) |
| Red/pr meat - Low |  |  |  |  |  |  | 3,955/2,251,273 | **0.95^*^** (0.91, 0.99) |
| White meat only |  |  |  |  |  |  | 116/67,669 | 1.00 (0.83, 1.20) |
| No meat/vegetarian |  |  |  |  |  |  | 35/18,769 | 1.21 (0.86, 1.70) |

HR (95% CI) from Cox proportional hazards models with statistically significant results in bold: ^*^ *P* < 0.05, ^†^ *P* < 0.01, ^‡^ *P* < 0.001; pr, processed

^1^ Subgroups are based on formal interaction tests showing significant effect modification (otherwise, cells are left blank); The interactions tested were each added separately to Model 5 as follows: diet^*^sex, diet^*^simple BMI category, diet^*^simple smoking status, diet^*^age group, diet^*^attained age.

^2^ Model 5 (fully adjusted) = mortality outcome ~ exposure + strata (age) + demographic factors demographics (sex [not adjusted in sex strata], race, education level, marital status, U.S. Census Bureau region) + lifestyle factors (12-level smoking status [not adjusted in smoking strata; replaced with years since quit (*Former* *smokers*) or cigarettes per day with years (*Current smokers*)], alcohol intake, physical activity, sleep) + health history (history of stroke [not adjusted in healthy cohort], high blood pressure, history of diabetes [not adjusted in healthy cohort], menopause status, hormone replacement therapy) + 6-level BMI (continuous BMI for BMI strata)

^3^ Exclusions were as follows: History of heart diseases, cancers (except for non-melanoma skin cancers), stroke, chronic respiratory conditions (emphysema, chronic bronchitis, or asthma), diabetes mellitus, chronic kidney diseases, chronic liver diseases.

^4^ Table does not present results for ever- and current smokers due to insufficient mortality case counts (i.e., ≤ 20 deaths in the no meat/vegetarian diet category).

Supplemental Table 8. Risk of all-cause mortality by diet categories overall and stratified by subgroups^1^ with results across statistical models^2^ in the sensitivity analyses including only long-term non-smokers^3^

|  | **Full analytic cohort**  *n =* 988,378 | | **Long-term non-smokers**  *n =* 513,135 | |
| --- | --- | --- | --- | --- |
| **Participants analyzed** | **Deaths/person-years** | **Fully adjusted model** | **Deaths/person-years** | **Fully adjusted model** |
| **ALL PARTICIPANTS** |  |  |  |  |
| Red/pr meat - High | 226,783/9,531,167 | *Reference* | 101,718/4,959,535 | *Reference* |
| Red/pr meat - Moderate | 190,140/7,929,461 | **0.99^‡^** (0.98, 0.99) | 91,102/4,281,596 | **0.99^*^** (0.98, 1.00) |
| Red/pr meat - Low | 201,402/7,597,227 | **0.98^‡^** (0.97, 0.98) | 102,316/4,208,254 | **0.99^†^** (0.98, 0.99) |
| White meat only | 5,495/206,327 | **0.96^†^** (0.93, 0.98) | 3,149/122,788 | 0.97 (0.94, 1.01) |
| No meat/vegetarian | 2,197/87,110 | 0.97 (0.93, 1.01) | 1,519/63,588 | 0.98 (0.94, 1.04) |
| **BMI CATEGORY** |  |  |  |  |
| **Healthy weight** | ***n =* 509,633** |  | ***n =* 269,479** |  |
| Red/pr meat - High | 100,512/4,692,919 | *Reference* | 44,899/2,517,335 | *Reference* |
| Red/pr meat - Moderate | 93,455/4,293,463 | **0.98^‡^** (0.97, 0.99) | 44,488/2,358,162 | **0.98^†^** (0.97, 1.00) |
| Red/pr meat - Low | 103,782/4,273,462 | **0.97^‡^** (0.96, 0.98) | 52,418/2,397,294 | **0.97^‡^** (0.96, 0.99) |
| White meat only | 3,187/133,098 | **0.95^†^** (0.91, 0.98) | 1,862/80,696 | 0.97 (0.93, 1.02) |
| No meat/vegetarian | 1,295/54,727 | 1.01 (0.95, 1.06) | 899/40,331 | 1.01 (0.95, 1.08) |
| **Overweight** | ***n =* 369,608** |  | ***n =* 183,822** |  |
| Red/pr meat - High | 93,246/3,674,405 | *Reference* | 40,221/1,807,569 | *Reference* |
| Red/pr meat - Moderate | 73,569/2,838,602 | 0.99 (0.98, 1.00) | 34,273/1,471,044 | 0.99 (0.98, 1.01) |
| Red/pr meat - Low | 74,814/2,616,677 | **0.99^*^** (0.98, 1.00) | 37,113/1,397,890 | 1.00 (0.98, 1.01) |
| White meat only | 1,785/58,516 | 0.97 (0.93, 1.02) | 975/33,136 | 0.97 (0.91, 1.04) |
| No meat/vegetarian | 702/25,259 | 0.94 (0.88, 1.02) | 485/18,041 | 0.99 (0.90, 1.08) |
| **Obesity** | ***n =* 109,137** |  | ***n =* 59,834** |  |
| Red/pr meat - High | 33,025/1,163,842 | *Reference* | 16,598/634,631 | *Reference* |
| Red/pr meat - Moderate | 23,116/797,396 | 1.01 (0.99, 1.02) | 12,341/452,389 | 1.01 (0.98, 1.03) |
| Red/pr meat - Low | 22,806/707,088 | 1.00 (0.98, 1.01) | 12,785/413,070 | 1.01 (0.99, 1.04) |
| White meat only | 523/14,713 | 0.99 (0.91, 1.08) | 312/8,955 | 0.97 (0.87, 1.09) |
| No meat/vegetarian | 200/7,124 | 0.87 (0.76, 1.00) | 135/5,217 | **0.80^*^** (0.68, 0.95) |
| **AGE GROUP** |  |  |  |  |
| **Less than 50 y** | ***n =* 257,856** |  | ***n =* 122,039** |  |
| Red/pr meat - High | 43,787/3,753,895 | *Reference* | 15,881/1,824,197 | *Reference* |
| Red/pr meat - Moderate | 30,157/2,785,951 | 1.00 (0.98, 1.01) | 11,410/1,379,905 | 1.00 (0.98, 1.02) |
| Red/pr meat - Low | 23,298/2,144,495 | 0.99 (0.98, 1.01) | 8,856/1,050,900 | 1.01 (0.99, 1.04) |
| White meat only | 525/56,641 | 0.97 (0.89, 1.06) | 222/28,759 | 1.02 (0.90, 1.17) |
| No meat/vegetarian | 266/28,825 | 1.09 (0.96, 1.23) | 145/19,317 | 1.01 (0.86, 1.19) |
| **50 to 64 y** | ***n =* 514,981** |  | ***n =* 260,655** |  |
| Red/pr meat - High | 135,375/4,887,174 | *Reference* | 60,101/2,585,422 | *Reference* |
| Red/pr meat - Moderate | 113,914/4,240,937 | **0.99^†^** (0.98, 1.00) | 53,329/2,325,595 | **0.99^*^** (0.97, 1.00) |
| Red/pr meat - Low | 113,628/4,188,521 | **0.97^‡^** (0.97, 0.98) | 54,839/2,324,564 | **0.98^†^** (0.97, 0.99) |
| White meat only | 2,791/108,441 | **0.94^‡^** (0.90, 0.97) | 1,509/64,551 | 0.97 (0.92, 1.02) |
| No meat/vegetarian | 993/40,492 | **0.94^*^** (0.88, 1.00) | 697/30,484 | 0.96 (0.89, 1.04) |
| **65 y or more** | ***n =* 215,541** |  | ***n =* 130,441** |  |
| Red/pr meat - High | 47,621/890,097 | *Reference* | 25,736/549,917 | *Reference* |
| Red/pr meat - Moderate | 46,069/902,573 | **0.98^†^** (0.97, 0.99) | 26,363/576,096 | 0.99 (0.97, 1.01) |
| Red/pr meat - Low | 64,476/1,264,210 | **0.97^‡^** (0.96, 0.98) | 38,621/832,789 | **0.98^*^** (0.97, 1.00) |
| White meat only | 2,179/41,245 | 0.99 (0.95, 1.03) | 1,418/29,477 | 0.97 (0.92, 1.03) |
| No meat/vegetarian | 938/17,793 | 0.98 (0.92, 1.05) | 677/13,788 | 1.00 (0.92, 1.08) |
| **ATTAINED AGE** |  |  |  |  |
| **Less than 80 y** | ***n =* 972,717** |  | ***n =* 501,178** |  |
| Red/pr meat - High | 106,100/7,818,882 | *Reference* | 36,470/3,914,738 | *Reference* |
| Red/pr meat - Moderate | 81,658/6,352,383 | **0.99^*^** (0.98, 1.00) | 29,951/3,293,676 | 0.99 (0.97, 1.00) |
| Red/pr meat - Low | 80,077/5,853,832 | **0.98^‡^** (0.97, 0.99) | 31,137/3,095,373 | 0.99 (0.97, 1.00) |
| White meat only | 2,102/155,546 | 1.00 (0.96, 1.05) | 959/88,209 | 1.02 (0.96, 1.09) |
| No meat/vegetarian | 809/66,214 | 1.02 (0.95, 1.10) | 463/46,969 | 1.02 (0.93, 1.12) |
| **80 y or more** | ***n =* 668,289** |  | ***n =* 389,228** |  |
| Red/pr meat - High | 120,683/1,712,284 | *Reference* | 65,248/1,044,797 | *Reference* |
| Red/pr meat - Moderate | 108,482/1,577,077 | **0.99^*^** (0.98, 1.00) | 61,151/987,920 | 0.99 (0.98, 1.00) |
| Red/pr meat - Low | 121,325/1,743,394 | **0.98^‡^** (0.97, 0.98) | 71,179/1,112,881 | **0.99^†^** (0.97, 1.00) |
| White meat only | 3,393/50,781 | **0.94^‡^** (0.91, 0.97) | 2,190/34,578 | **0.95^*^** (0.91, 1.00) |
| No meat/vegetarian | 1,388/20,896 | 0.95 (0.90, 1.00) | 1,056/16,620 | 0.97 (0.91, 1.03) |

HR (95% CI) from Cox proportional hazards models with statistically significant results in bold: ^*^ *P* < 0.05, ^†^ *P* < 0.01, ^‡^ *P* < 0.001; pr, processed

^1^ Subgroups are based on formal interaction tests showing significant effect modification (otherwise, cells are left blank); The interactions tested were each added separately to the “All non-smokers” Model 5 as follows: diet^*^sex, diet^*^simple BMI category, diet^*^age group, diet^*^attained age.

^2^ Statistical models: **Model 1** = mortality outcome ~ exposure + strata (age); **Model 2** = Model 1 + demographic factors demographics (sex, race, education level, marital status, U.S. Census Bureau region); **Model 3** = Model 2 + lifestyle factors (alcohol intake, physical activity, sleep); **Model 4** = Model 3 + health history (number of comorbidities, high blood pressure); **Model 5 (fully adjusted)** = 4 + 6-level BMI (continuous BMI for BMI strata)

^3^ Never-smokers and former smokers with 20 years or more since quitting

Supplemental Table 9. Risk of all-cause and cause-specific mortality in the full analytic cohort (*n =* 988,378) with results by meat type and across statistical models in the post-hoc exploratory analysis^1^

| **Cause of mortality** | **Deaths/person-years** | **Model 1**  Age | **Model 2**  Demographics | **Model 3**  Lifestyle | **Model 4**  Health | **Model 5**  BMI |
| --- | --- | --- | --- | --- | --- | --- |
| **All causes** |  |  |  |  |  |  |
| Red/pr & white meat | 609,533/24,773,573 | *Reference* | *Reference* | *Reference* | *Reference* | *Reference* |
| Red/pr meat only | 8,792/284,281 | **1.26^‡^** (1.24, 1.29) | **1.16^‡^** (1.13, 1.18) | **1.10^‡^** (1.08, 1.12) | **1.11^‡^** (1.08, 1.13) | **1.11^‡^** (1.08, 1.13) |
| White meat only | 5,495/206,327 | **0.91^‡^** (0.89, 0.93) | **0.94^‡^** (0.91, 0.96) | **0.97^*^** (0.94, 0.99) | **0.96^†^** (0.93, 0.98) | **0.97^*^** (0.95, 1.00) |
| No meat/vegetarian | 2,197/87,110 | **0.89^‡^** (0.85, 0.92) | **0.91^‡^** (0.87, 0.94) | **0.96^*^** (0.92, 1.00) | 0.98 (0.94, 1.02) | 0.99 (0.95, 1.03) |
| **Diseases of the heart** |  |  |  |  |  |  |
| Red/pr & white meat | 173,444/24,773,573 | *Reference* | *Reference* | *Reference* | *Reference* | *Reference* |
| Red/pr meat only | 2,593/284,281 | **1.27^‡^** (1.22, 1.32) | **1.13^‡^** (1.09, 1.18) | **1.07^‡^** (1.03, 1.11) | **1.10^‡^** (1.06, 1.14) | **1.11^‡^** (1.06, 1.15) |
| White meat only | 1,754/206,327 | 0.96 (0.92, 1.01) | 1.00 (0.96, 1.05) | 1.01 (0.96, 1.06) | 0.97 (0.92, 1.02) | 1.00 (0.95, 1.05) |
| No meat/vegetarian | 678/87,110 | **0.89^†^** (0.83, 0.96) | 0.93 (0.86, 1.00) | 0.94 (0.87, 1.02) | 0.98 (0.91, 1.06) | 1.00 (0.93, 1.08) |
| **Cancers** |  |  |  |  |  |  |
| Red/pr & white meat | 157,956/24,773,573 | *Reference* | *Reference* | *Reference* | *Reference* | *Reference* |
| Red/pr meat only | 2,244/284,281 | **1.23^‡^** (1.18, 1.28) | **1.13^‡^** (1.08, 1.17) | **1.07^†^** (1.03, 1.12) | **1.07^†^** (1.03, 1.12) | **1.07^‡^** (1.03, 1.12) |
| White meat only | 1,280/206,327 | **0.88^‡^** (0.83, 0.93) | **0.91^†^** (0.86, 0.96) | 0.97 (0.92, 1.03) | 0.97 (0.92, 1.03) | 0.98 (0.93, 1.04) |
| No meat/vegetarian | 512/87,110 | **0.85^‡^** (0.78, 0.93) | **0.88^†^** (0.81, 0.96) | 1.00 (0.91, 1.09) | 0.99 (0.91, 1.08) | 1.00 (0.92, 1.09) |
| **Stroke** |  |  |  |  |  |  |
| Red/pr & white meat | 42,545/24,773,573 | *Reference* | *Reference* | *Reference* | *Reference* | *Reference* |
| Red/pr meat only | 585/284,281 | **1.18^‡^** (1.09, 1.28) | **1.13^†^** (1.04, 1.23) | **1.09^*^** (1.01, 1.19) | **1.10^*^** (1.02, 1.20) | **1.10^*^** (1.02, 1.20) |
| White meat only | 420/206,327 | 0.92 (0.84, 1.01) | 0.93 (0.85, 1.03) | 0.94 (0.86, 1.04) | 0.95 (0.86, 1.04) | 0.95 (0.86, 1.04) |
| No meat/vegetarian | 200/87,110 | 1.05 (0.92, 1.21) | 1.05 (0.91, 1.21) | 1.07 (0.93, 1.23) | 1.11 (0.96, 1.27) | 1.10 (0.96, 1.27) |

HR (95% CI) from Cox proportional hazards models with statistically significant results in bold: ^*^ *P* < 0.05, ^†^ *P* < 0.01, ^‡^ *P* < 0.001; pr, processed

^1^ Statistical models: **Model 1** = mortality outcome ~ exposure + strata (age); **Model 2** = Model 1 + demographic factors demographics (sex, race, education level, marital status, U.S. Census Bureau region); **Model 3** = Model 2 + lifestyle factors (12-level smoking status, alcohol intake, physical activity, sleep); **Model 4** = Model 3 + health history (Unique covariates for each mortality outcome were as follows. *All causes*: number of comorbidities, high blood pressure; *Heart diseases:* history of heart disease, high blood pressure, menopause status, hormone replacement therapy; *Cancers*: history of cancer, family history of cancer, history of diabetes, menopause status, hormone replacement therapy; *Stroke*: history of stroke, high blood pressure, history of diabetes, menopause status, hormone replacement therapy); **Model 5** (fully adjusted) = Model 4 + 6-level BMI
